# The intricate cellular ecosystem of human peripheral veins as revealed by single-cell transcriptomic analysis

**DOI:** 10.1371/journal.pone.0296264

**Published:** 2024-01-11

**Authors:** Miguel G. Rojas, Zachary M. Zigmond, Simone Pereira-Simon, Nieves Santos Falcon, Maya Suresh Kumar, Filipe F. Stoyell-Conti, Christina Kosanovic, Anthony J. Griswold, Alghidak Salama, Xiaofeng Yang, Marwan Tabbara, Roberto I. Vazquez-Padron, Laisel Martinez

**Affiliations:** 1 DeWitt Daughtry Family Department of Surgery, Leonard M. Miller School of Medicine, University of Miami, Miami, Florida, United States of America; 2 Bruce W. Carter Veterans Affairs Medical Center, Miami, Florida, United States of America; 3 John P. Hussman Institute for Human Genomics, Leonard M. Miller School of Medicine, University of Miami, Miami, Florida, United States of America; 4 Department of Cardiovascular Sciences, Lewis Katz School of Medicine at Temple University, Philadelphia, Pennsylvania, United States of America; University of Bonn, Institute of Experimental Hematology and Transfusion Medicine, GERMANY

## Abstract

The venous system has been historically understudied despite its critical roles in blood distribution, heart function, and systemic immunity. This study dissects the microanatomy of upper arm veins at the single cell level, and how it relates to wall structure, remodeling processes, and inflammatory responses to injury. We applied single-cell RNA sequencing to 4 non-diseased human veins (3 basilic, 1 cephalic) obtained from organ donors, followed by bioinformatic and histological analyses. Unsupervised clustering of 20,006 cells revealed a complex ecosystem of endothelial cell (EC) types, smooth muscle cell (SMCs) and pericytes, various types of fibroblasts, and immune cell populations. The venous endothelium showed significant upregulation of cell adhesion genes, with arteriovenous zonation EC phenotypes highlighting the heterogeneity of vasa vasorum (VV) microvessels. Venous SMCs had atypical contractile phenotypes and showed widespread localization in the intima and media. *MYH11*^+^*DES*^*lo*^ SMCs were transcriptionally associated with negative regulation of contraction and pro-inflammatory gene expression. *MYH11*^+^*DES*^*hi*^ SMCs showed significant upregulation of extracellular matrix genes and pro-migratory mediators. Venous fibroblasts ranging from secretory to myofibroblastic phenotypes were 4X more abundant than SMCs and widely distributed throughout the wall. Fibroblast-derived angiopoietin-like factors were identified as versatile signaling hubs to regulate angiogenesis and SMC proliferation. An abundant monocyte/macrophage population was detected and confirmed by histology, including pro-inflammatory and homeostatic phenotypes, with cell counts positively correlated with age. Ligand-receptor interactome networks identified the venous endothelium in the main lumen and the VV as a niche for monocyte recruitment and infiltration. This study underscores the transcriptional uniqueness of venous cells and their relevance for vascular inflammation and remodeling processes. Findings from this study may be relevant for molecular investigations of upper arm veins used for vascular access creation, where single-cell analyses of cell composition and phenotypes are currently lacking.

## Introduction

The systemic venous system is an often-underappreciated component of the cardiovascular system, with critical roles in blood distribution, heart function, and even systemic immunity. Research of vein biology has historically relied on extrapolations from arterial biology, despite differences in structure, functions, hemodynamics, and propensity to diseases. In the last two decades, transcriptomic studies have provided in-depth characterizations of molecular processes in healthy and diseased human arteries [1–5], but little attention has been paid to human veins [6–8]. This paucity of data limits our understanding of venous pathologies and of cells populating half of the circulatory system.

Recently, single-cell RNA sequencing has revealed an unappreciated level of cellular heterogeneity, identified unknown cell types, and elucidated cellular differentiation and interaction pathways associated with atherosclerosis and aortic aneurysms [2, 4, 5, 9–11]. However, the perturbations in cellular ecology underlying venous diseases such as thrombophlebitis, deep vein thrombosis, and chronic vein insufficiency remain unknown. Peripheral veins, in particular, are commonly affected by stenotic remodeling after their use for vein grafts or creation of arteriovenous fistulas (AVF) for hemodialysis [12–14]. The frequency of these complications, prevalence of chronic venous diseases, and their significant contribution to patient mortality and morbidity stress the need for high-throughput discovery methods to dissect cellular dynamics within the healthy and diseased venous wall.

Histological inspection of human saphenous and upper extremity veins shows structural wall irregularities regardless of age or the presence of common comorbidities [15–20]. Frequent characteristics include medial atrophy, disorganized smooth muscle cells (SMCs), intimal hyperplasia (IH), and abundant interstitial extracellular matrix (ECM) suggesting a natural tendency to wall fibrosis [14, 15, 17–22]. The absence or thinning of the internal elastic lamina (IEL) and the abundance of fragmented elastic fibers scattered across the wall may have important implications for cell migration and inflammation [16, 22, 23]. The interstitial space between SMC bundles seems to be populated by cells of unknown lineages [16]. Endothelial defects and intramural vascularization that ranges from protective to pathogenic have also been described [18, 20, 24–27]. Despite all available knowledge of venous histology, how these characteristics relate to normal physiology or disease is unclear. Thus, as in studies of arteries, single-cell analyses hold great promise to understand the relationship between those unique structural characteristic and venous function.

In this study, we generate a detailed single-cell transcriptomic atlas of upper extremity veins with histological validations. We reveal a complex ecosystem of ECs with various arteriovenous zonation phenotypes, and an interactive network of discrete SMC populations, different types of fibroblasts, and abundant immune cells in the venous wall. Our analyses support a role for ECs in the main lumen and vasa vasorum (VV) venules, SMCs, and pericytes in promoting immune cell recruitment and infiltration. We also support a protagonist role of fibroblasts and atypical SMCs in angiogenesis and wall remodeling. Overall, this work constitutes an essential foundation for the study of veins commonly used for vascular access creation and the peripheral venous system in general.

## Results

### Single-cell RNA sequencing of upper arm veins

**Fig 1:**
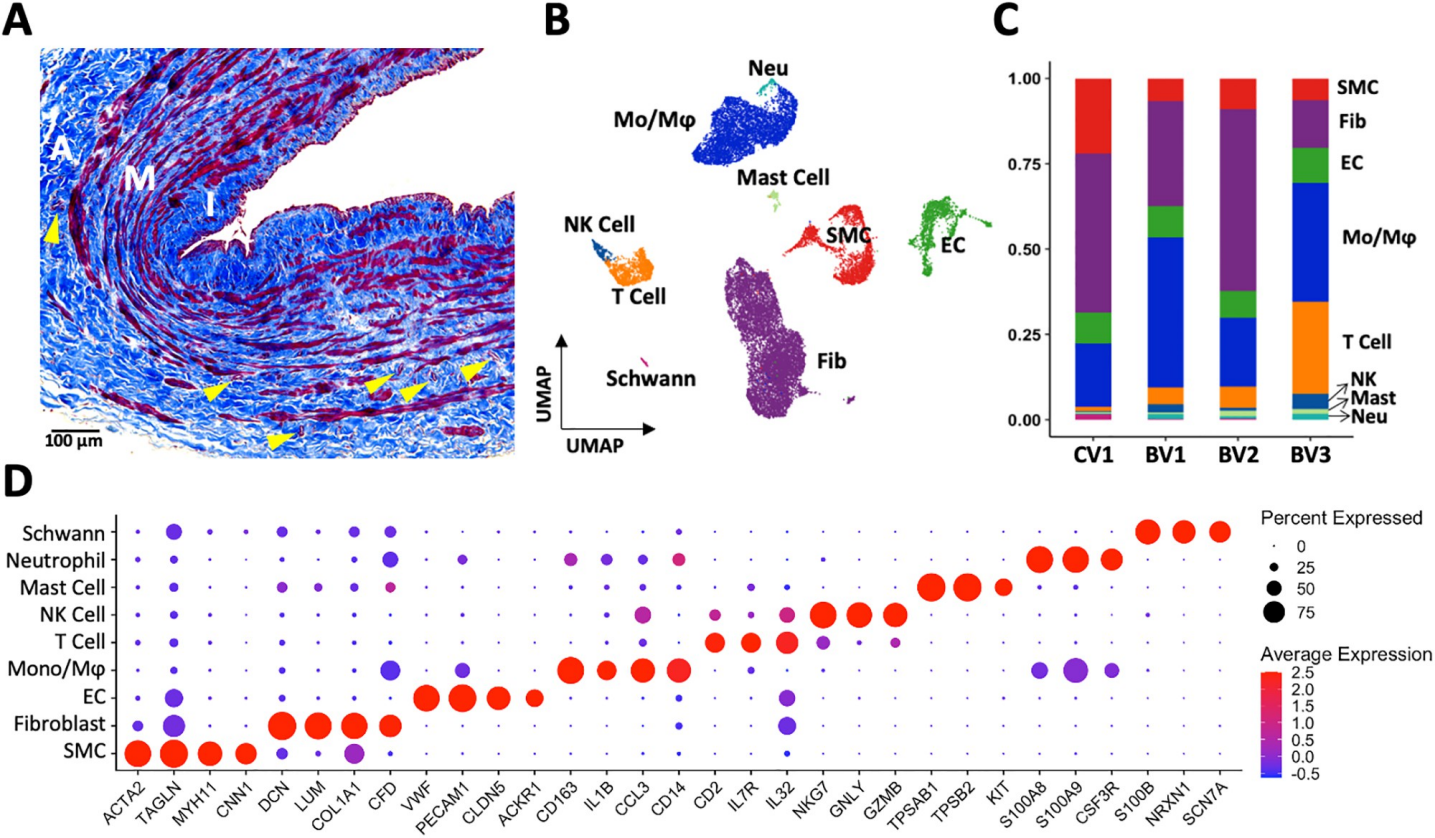
Wall structure and cell composition of upper arm veins. **A)** Representative Masson’s trichrome stained cross-section of a basilic vein, indicating the intimal (I), medial (M), and adventitial (A) layers. Cells are stained in red while extracellular matrix appears in blue. Vasa vasorum are indicated by yellow arrowheads. **B-C)** Uniform manifold approximation and projection (UMAP) plot of 20,006 cells isolated from 4 veins (1 cephalic [CV1], 3 basilic [BV1-3]) and proportion of cell types per vein sample. **D)** Markers used for cell cluster identification.

A representative cross-section of a medium-size peripheral vein is characterized by 1) low to moderate IH, 2) a thin or fragmented IEL, 3) circular or diagonally oriented cells between the IEL and longitudinal smooth muscle fibers, 4) medial SMC bundles intercalated in abundant ECM, 5) fragmented elastic fibers in the adventitia, and 6) numerous VV in the outer layers of the vessel (Fig 1A, S1 Fig). To explore this microanatomy in further detail, cell suspensions were obtained from three basilic and one cephalic vein collected from the upper arms of organ donors and analyzed using single-cell RNA sequencing (S2 Fig). The group of donors was highly variable in terms of age (26–70 years), sex (50:50), and ethnic distribution (2 whites, 1 Hispanic, 1 black) (S1 Table). Causes of death included head trauma (26F and 64M), cardiovascular (38F), and cerebrovascular events (70M). One donor had history of hypertension (38F) and none had diabetes. We did not observe significant differences in morphometry between upper arm basilic and cephalic veins (S1 Fig), in agreement with published data [15].

Fig 1B presents a coarse clustering map of 20,006 cells, with proportions of cell populations separated by sample identity (Fig 1C, S2C Fig). Fibroblasts (14–47%) and monocyte/macrophages (18–44%) had the highest cell proportions in individual samples, followed by ECs (8–10%), SMCs (7–22%), and T cells (1–26%; Fig 1C). The top Seurat markers for SMC identification were contractile genes (*ACTA2*, *TAGLN*, *MYH11*, *CNN1*), extracellular matrix genes and complement factors in fibroblasts (*DCN*, *LUM*, *COL1A1*, *CFD*), cell adhesion molecules and tight junction proteins in ECs (*VWF*, *PECAM1*, *CLDN5*, *ACKR1*), and various cell surface receptors and secreted factors for immune cell populations (monocytes/macrophages, *CD163*, *IL1B*, *CCL3*, *CD14*; neutrophils, *S100A8/9*, *CSF3R*; T cells, *CD2*, *IL7R*, *IL32*; NK cells, *NKG7*, *GNLY*, *GZMB*; mast cells, *TPSAB1*, *TPSB2*, *KIT*) (Fig 1D). Lastly, we found a small Schwann cell population defined by *S100B*, *NRXN1*, and *SCN7A*, likely originating from periadventitial neurovascular tissue (Fig 1D). We then proceeded to characterize these main populations in detail, focusing on subpopulation gene expression profiles, localization, and cell-to-cell communication networks.

### A pro-inflammatory endothelium in upper arm veins

**Fig 2:**
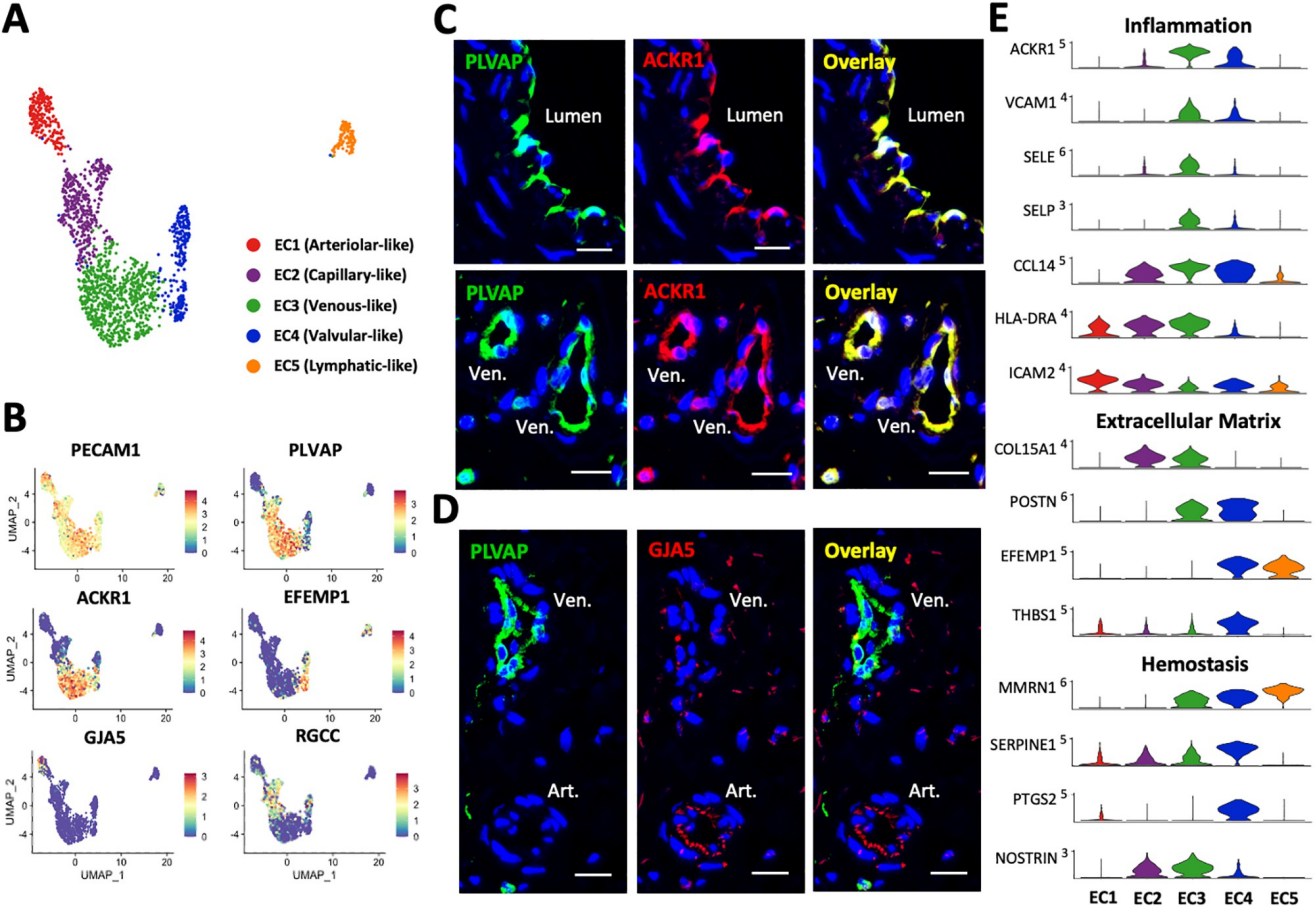
Endothelial cells (EC) in upper arm veins. **A)** Focused UMAP of 1,695 ECs isolated from upper arm veins. **B)** Feature plots indicating normalized expression levels of markers characteristic of the 5 EC populations. **C-D)** Identification of venous (PLVAP^+^ACKR1^+^) and arteriolar (PLVAP^-^GJA5^+^) ECs in the main lumen of veins and vasa vasorum by immunofluorescence. Ven. = venule; Art. = arteriole. Scale bars represent 20 μm in all panels. **E)** Violin plot representation of expression differences among the 5 EC populations.

Endothelial cells are one of the most heterogenous populations in peripheral veins due to the presence of valves and the abundant VV irrigating the venous wall. The proportion of ECs ranged from 7.8 to 10.1% across the 4 veins analyzed (Fig 1C), for a total of 1,695 cells out of 20,006 in the focused uniform manifold approximation and projection (UMAP) plot (Fig 2A). These subdivided into five groups corresponding to arteriovenous zonation phenotypes annotated elsewhere [6, 28–30]: EC1 [arteriolar-like], 215 cells; EC2 [capillary-like], 400; EC3 [venous-like], 712; EC4 [valvular-like], 257; and EC5 [lymphatic-like], 111 (Fig 2). Canonical markers in common among all EC subsets were *CDH5*, *CLDN5*, *ERG*, *PECAM1*, and *VWF* (Fig 1D), while markers characterizing each population are presented in S3A Fig and Table 1.

**Table 1:** Marker genes for cell cluster and subcluster identification and their predicted functions in upper arm veins.

Cluster/Subcluster*	Marker Genes	Classification/Predicted Function
**Endothelial cells**	*VWF*, *PECAM1*, *CDH5*	Pan-EC markers
EC1–Arteriolar	*NOTCH4*, *SOX17*, *HEY1*	Arterial EC differentiation
	*EFNB2*, *SEMA3G*	Angiogenesis
	*NEBL*, *TSPAN2*	Cytoskeleton organization
	*GJA5*	Gap junction
EC2–Capillary	*CA4*, *FMO2*, *RBP7*	pH and redox homeostasis
	*CD300LG*	Leukocyte binding/rolling
	*CD36*	Fatty acid uptake
	*RGCC*	Cell cycle regulation
EC3–Venous	*NR2F2*	Venous EC differentiation
	*PLVAP*, *AQP1*	Permeability
	*ACKR1*	Chemokine transcytosis
	*ICAM1*, *VCAM1*, *SELE*, *SELP*	Leukocyte adhesion
EC4–Valvular	*FOXC2*	Valvular EC differentiation
	*CRTAC1*, *EFEMP1*, *POSTN*, *HAPLN3*	Extracellular matrix (ECM)
	*BMP2*, *GDF7*	ECM synthesis
	*SERPINE1*, *PTGS2*	Hemostasis
EC5–Lymphatic	*FOXC2*, *NR2F1*, *PROX1*	Lymphatic EC differentiation
	*LYVE1*, *PDPN*, *RELN*	Leukocyte trafficking
**Smooth muscle cells**	*ACTA2*, *SRF*, *MRTFA*, *MRTFB*	Pan-SMC/pericyte markers
SMC1–Desmin^high^	*MYH11*, *CALD1*, *TAGLN*	SMC contraction
	*DES*, *CNN1*, *MYLK*, *ADRA2C*	Regulation of contraction
	*FN1*, *COL1A2*, *COL3A1*, *VCAN*	ECM
	*ITGA5*, *ITGA8*	ECM binding
SMC2–Desmin^low^	*MYH11*, *CALD1*, *TAGLN*	SMC contraction
	*PLN*, *SORBS2*, *NET1*, *RERGL*, *HRH2*, *ADRA2A*	Regulation of contraction
	*NDUFA4L2*	Low-oxygen sensing
	*PGF*	Angiogenesis; inflammation
SMC3–Pericyte	*CD36*, *APOE*, *STEAP4*, *FABP4*	Fatty acid uptake/metabolism
	*CYGB*	Oxygen diffusion
	*EDNRB*	Vasoreactivity
	*THY1*	Stemness; leukocyte infiltration
**Fibroblasts**	*PDGFRA*, *LUM*, *C1R*, *DCN*, *THY1*	Pan-fibroblast markers
Fib1–Activated	*COL1A1*, *COL1A2*, *COL3A1*, *BGN*, *POSTN*, *FN1*	ECM; cell migration
	*ACTA2*, *TAGLN*, *NREP*	Myofibroblastic transition
	*NDUFA4L2*	Low-oxygen sensing
	*F2R*	Hemostasis
	*ITGA10*	ECM binding
Fib2–Maintenance	*COL1A1*, *COL1A2*, *COL3A1*, *BGN*	ECM
	*NDUFA4L2*	Low-oxygen sensing
Fib3–Secretory	*C1S*, *C3*, *CFD*	Complement factors
	*CD34*, *CXCL14*, *SFRP1*, *SVEP1*	Regulation of angiogenesis
	*APOD*, *IGF1*, *IGFBP5*, *PLA2G2A*, *PLPP3*, *GPX3*, *MGST1*	Lipid metabolism
	*SCARA5*	Ferritin-iron uptake; adipocytic commitment
**Monocyte/macrophages**	*CD163*, *CD14*, *FCGR3A*, *MRC1*	Pan-mo/mφ markers
Mo/Mφ1–IL1B^+^	*IL1B*, *NLRP3*, *EREG*, *CCL20*, *TNF*	Inflammation
Mo/Mφ2–IL1B^-^	C1QA, C1QB, C1QC, VSIG4	Inflammation resolution
	SELENOP	Redox homeostasis
	FOLR2	Folate uptake

There is a continuum of transcriptional regulation across the five EC populations as demonstrated using pseudotime trajectory analysis (S3B Fig). From arteriolar-like (EC1) to lymphatic-like ECs (EC5), there is a decreasing expression gradient in arterial differentiation factors such as *NOTCH4*, *SOX17*, and *HEY1* (Moran’s I = 0.34 to 0.43, padj<0.001) and the opposite trend in the venous differentiation factor *NR2F2* (Moran’s I = 0.43). Levels of *FOXC2*, which is linked to primary venous valve failure and lymphedema when mutated [31], also increase from valvular to lymphatic ECs (Moran’s I = 0.49; S3B Fig). Factors upregulated in venous ECs with respect to the other populations include *ZNF385D* and *MRTFB* (**S1 File in** S1 Data). Bidirectional signaling between the *EPHB4* receptor in venous ECs and ephrin B2 ligand (*EFNB2*) in arterial ECs is critical for vasculature development in the embryo [32, 33], and thought to be relevant for remodeling of veins after arteriovenous anastomosis [34]. In our dataset, the expression pattern of *EFNB2* does show a decreasing gradient from arteriolar- to venous-like ECs, but *EPHB4* does not discriminate among the different EC subclusters (S3B Fig). This latter observation agrees with IF staining of arteries and veins from adult tissues [35]. As expected, capillary-like ECs co-express high levels of both arterial and venous differentiation factors, such as *NOTCH4* and *NR2F2* (S3B Fig), as well as other genes characteristic of arteriolar (e.g., *ADGRF5*, *CXCL12*, *IGFBP3*) and venous populations (e.g., *COL15A1*, *NOSTRIN*, *PLVAP*) (**S1 File in** S1 Data). This overlap may reflect the size spectrum of microvessels in capillary networks [28, 36], but we lack the resolution for better definition.

The most abundant EC subpopulation in human peripheral veins is the venous-like cluster that lines the main lumen as well as VV venules (Fig 2A–2C, S4 Fig). These are transcriptionally defined by *ACKR1*, *SELE*, *SELP*, *TLL1*, *VCAM1*, and *ZNF385D* (S3A Fig) and are positive for PLVAP and ACKR1 by immunofluorescence (IF) (Fig 2C and 2D, S4A Fig). Localization of ACKR1 in the main lumen of veins demonstrates that this marker is not restricted to post-capillary venules as reported in mice [37]. The transcriptional program of venous ECs highlights unique hemostatic, cell adhesive, and inflammatory properties of the venous endothelium. High expression of plasmalemma vesicle-associated protein (*PLVAP*) and aquaporin 1 (*AQP1*) with respect to other subsets illustrates an environment of increased vascular permeability [38]. In addition, upregulated levels of adhesion molecules *ACKR1*, *ICAM1*, *VCAM1*, *SELE*, and *SELP* showcase these cells as a primed docking site for leukocyte infiltration, as predicted by gene ontology (GO) pathways (Fig 2E, S5A Fig). The vast majority of venous ECs are high expressors of *CCL14* (≥3 normalized copies in a scale of 0–5), a known activator of monocytes along with 188 (26.4%) and 445 out of 712 (62.5%) cells that show upregulation of *IL6* and *IL1R1* (≥1 normalized copy, 0–4 scale for both), respectively, likely indicating inflammatory activation. Upregulation of the nitric oxide synthase trafficker *NOSTRIN* in venous-like and capillary-like cells compared to the rest of ECs (Fig 2E) suggests that eNOS is preferentially located away from the membrane, with important consequences for hemostasis, vasoreactivity, and inflammation. In addition, the abundance of *EPB41L3* and *COL15A1* in venous ECs suggests a low predisposition for endothelial-mesenchymal transformation due to SNAI1 inhibition (S3C Fig) [39, 40].

While smaller in cell count, the arteriolar-like and capillary-like EC subclusters are also important contributors to the VV. Arteriolar ECs cells are characterized by expression of *GJA5*, *HEY1*, *ICAM2*, *NEBL*, *PLLP*, *SEMA3G*, *SYT1*, and *TSPAN2*. These cells stained negative for PLVAP while positive for GJA5 (Fig 2D, S4B Fig). Interestingly, they also showed upregulation of ICAM2 compared to other EC types, and in contrast with ICAM1 and the rest of the adhesion molecules which show higher levels in venous ECs (Fig 2E, **S1 File in** S1 Data). Expression of ICAM2 is thought to be a characteristic of a resting endothelium, where it can negatively regulate ICAM1-mediated transendothelial migration of leukocytes [41]. The lower levels of ICAM2 in venous ECs further supports their protagonist role in leukocyte infiltration and higher pro-inflammatory potential. Capillary-like ECs, in turn, are defined by *CA4*, *CD300LG*, *CD36*, *FCN3*, *FMO2*, *H19*, *RBP7*, *RGCC*, and *TCF15*, and likely correspond to the vast capillary network [24].

Valvular-like ECs have a distinct transcriptional profile from the non-valvular venous population, characterized by high expression of *ANGPTL4*, *BMP2*, *CLU*, *CRTAC1*, *EFEMP1*, *HHIP*, *PTGS2*, and *SERPINE1*. The transcriptional program in this subcluster is directed at ECM production (Fig 2E, S5A Fig), including extracellular proteins *CRTAC1*, *EFEMP1*, *HAPLN3*, *POSTN*, and *THBS1*, and regulators of ECM production and cell-ECM interactions such as *BMP2*, *GDF7*, and *FBLIM1*. Unlike the rest of the ECs, the valvular endothelium has low expression of HLA class II genes (*HLA-DRA*, *HLA-DRB1*, *HLA-DPA1*, etc.; Fig 2E, **S1 File in** S1 Data) or the invariant chain *CD74*, indicating a more immunologically neutral luminal surface. Importantly, valvular ECs seem more thromboresistant than the rest of the EC populations, since they overexpress *PTGS2* (also known as COX-2), the rate-limiting enzyme for prostacyclin biosynthesis (Fig 2E). Lastly, markers for lymphatic-like ECs include *CCL21*, *LYVE1*, *NR2F1*, *PDPN*, *PKHD1L1*, *PROX1*, *SEMA3A*, and *RELN* (S3A Fig), likely originating from lymphatic adventitial/periadventitial microvessels.

### Intimal and medial SMCs: More similarities than differences

**Fig 3:**
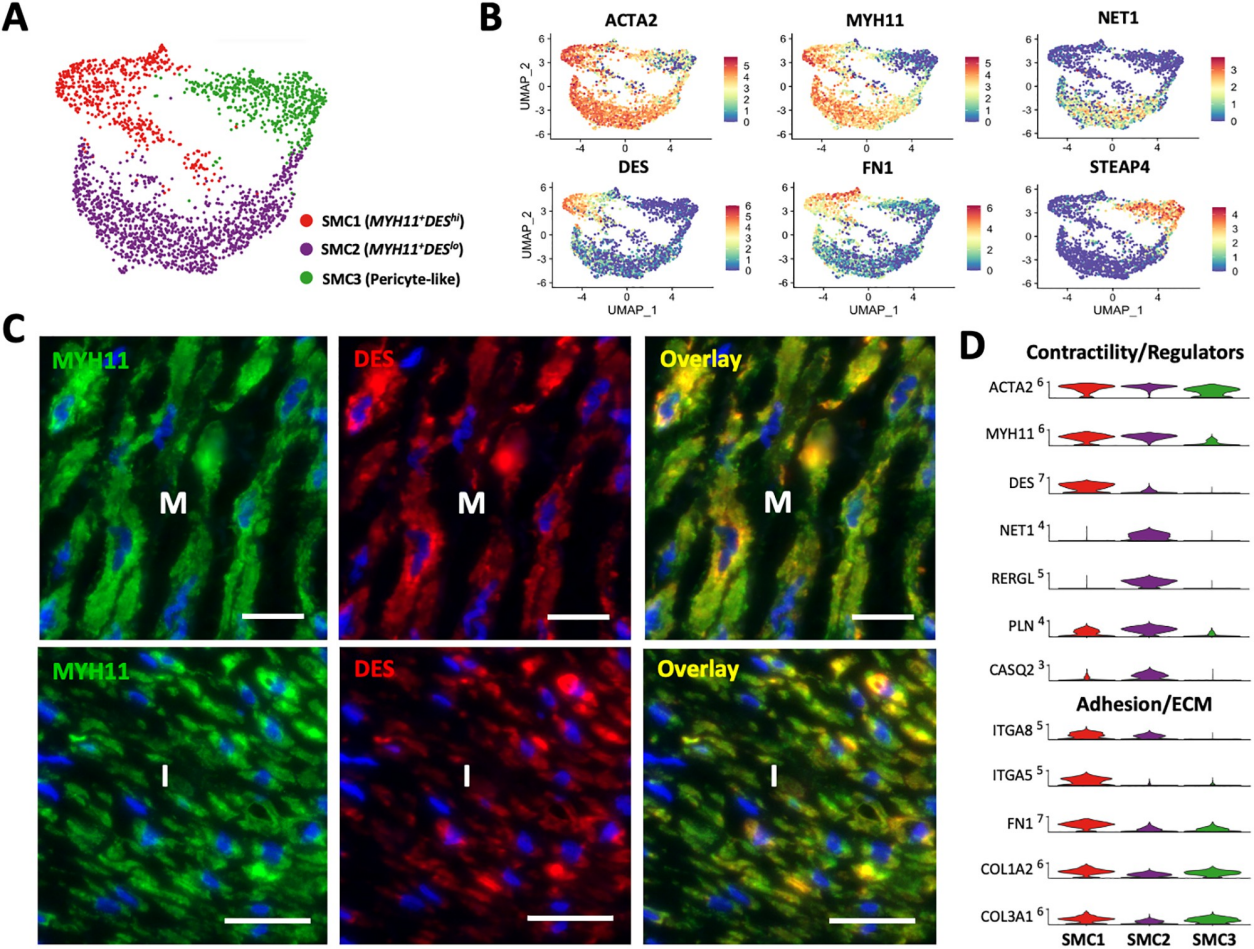
Smooth muscle cells (SMC) and pericytes in upper arm veins. **A)** Focused UMAP of 2,298 SMCs isolated from upper arm veins. **B)** Feature plots indicating normalized expression levels of markers characteristic of the 3 SMC populations. **C)** Localization of SMC1 (*MYH11*^+^*DES*^*hi*^) and SMC2 (*MYH11*^+^*DES*^*lo*^) cells in the intima (I) and media (M) by immunofluorescence. Scale bars represent 20 μm in all panels. **D)** Violin plot representation of expression differences among the 3 SMC populations.

Smooth muscle cells represent 6.6 to 21.9% of cells in individual samples (Fig 1C) and a total of 2,298 cells in the focused UMAP (Fig 3A). Two main subpopulations of SMCs (*MYH11*^+^*DES*^*hi*^ SMC1, 532 cells; *MYH11*^+^*DES*^*lo*^ SMC2, 1248) and a cluster of pericyte-like cells (SMC3, 518 cells) were detected. The SMC1 and SMC2 clusters share transcriptional features in common that reflect a fully differentiated contractile phenotype. These include high expression of the contractile markers *ACTA2*, *MYH11*, *CALD1*, and *TAGLN*; contractile accessory proteins *TPM1*, *TPM2*, and *PPP1R14A*; actin polymerization and depolymerization factors *DSTN* and *LMOD1*; and moderate to high expression of the main TFs and cofactors responsible for these characteristics (*SRF*, *MYOCD*, *MRTFA*, *MRTFB*, *CSRP1*) (Fig 3, S6A and S6B Fig). The small number of differentially expressed genes between the SMC1 and SMC2 subclusters suggests that they represent distinct transcriptional states of the same population (S6A Fig). A summary of marker genes identifying SMC subclusters is presented in Table 1.

Immunofluorescence analyses confirmed the widespread localization of MYH11-positive SMCs in the intima and media. However, both venous SMC subclusters displayed transcriptional characteristics that could be associated with a potential for phenotypic modulation. For instance, the SMC1 population has transcriptional features associated with both increased contractility and enhanced migratory potential, a synthetic characteristic (Fig 3B–3D). Cells in this cluster show increased expression of the contractile markers *DES*, *CNN1*, *MYLK*, and the cold-induced adrenergic receptor *ADRA2C* (Fig 3B, S6A Fig) [42–44]. In parallel, they demonstrate significant upregulation of the β1 integrin subunit *ITGA5* and its ligand fibronectin (*FN1*), the F-actin stabilizing protein *PALLD* [45], and the actin bundling protein *SYNPO2*, which further stabilizes lamellipodia and actin projections required for migration [46]. Upregulation of *ITGA5* is associated with a synthetic phenotype in arterial SMCs, as well as connexin 43 (*GJA1*) and versican (*VCAN*), which are also upregulated in the *MYH11*^+^*DES*^*hi*^ SMC1 cluster (**S3 File in** S1 Data) [44, 47]. Our analyses reveal that *ITGA5*^*hi*^ and *DES*^*hi*^ SMCs inhabit the intima and media irrespective of spindle-like or rounded morphologies (Fig 3C, S7A and S7B Fig). Upregulation of *FN1*, *COL1A2*, and *COL3A1* in the SMC1 cluster may also explain the extensive deposition of ECM in the media of veins (Fig 3D, S1B Fig) and propensity of these cells for phenotypic modulation [48, 49].

Interestingly, *MYH11*^+^*DES*^*lo*^ SMC2 cells show increased expression of distinct regulators of the cell cytoskeleton and contractile machinery, such as the sarcoplasmic reticulum Ca-ATPase inhibitor *PLN*, the cytoskeletal adaptor protein *SORBS2*, the Rho guanine exchange factor (and RhoA activating protein) *NET1*, and the GDP/GTP binding protein *RERGL* [42] (Fig 3D, S6A Fig). Activation of RhoA signaling can promote both the contractile and synthetic phenotypes depending on the ECM [50]. Enrichment of the laminin receptor *BCAM* in SMC2 cells may contribute to the differentiated SMC phenotype by enhancing interactions with the basement membrane [51, 52]. In addition, highly expressed metalloproteinases such as *ADAMTS4* and *ADAMTS9* also allow focal adhesion turnover and control cell proliferation [53]. Gene ontology analyses predict a suppression of calcium ion transport due to upregulation of *PLN* and *CASQ2*, which may affect contractility (S5B Fig). A balance in expression of negative and positive regulators of smooth muscle contraction such as *HRH2* and *ADRA2A*, respectively, further support this scenario. Transcriptional regulators with higher levels in this population include the SRF cofactor *CSRP2*, *NOTCH3*, *PPARG* and its co-stimulatory factor *ADIRF*, and the myogenic differentiation gene *MUSTN1* (S6B Fig, **S3 File in** S1 Data) [54]. A higher proportion of SMC2 cells presents upregulation of *IL6* (≥1 normalized copy) compared to the SMC1 cluster (240/1248, 19.2% vs. 21/532, 3.9%, respectively), indicating increased pro-inflammatory potential. Furthermore, upregulation of mitochondrial protein *NDUFA4L2* may represent an advantageous metabolic adaptation to low oxygen tension [55]. Immuno-localization analyses demonstrate that SMC2 cells co-exist in the intima and media with the SMC1 population, further supporting a migration-permissive environment in the wall irrespective of phenotypic characteristics. Cell-to-cell interactome analysis identified SMC2 cells as the main producers of placental growth factor (*PGF*) in the vein, with predicted paracrine effects on *FLT1* (VEGFR1)-expressing ECs and monocytes/macrophages (S6C Fig). Triggers of PGF secretion by SMCs include angiotensin II [56] and EC-derived reactive oxygen species in response to shear stress [57]. PGF is an angiogenic factor that promotes monocyte activation and recruitment to the endothelium [58–60].

Pericytes are supportive cells embedded within the endothelial basement membrane of VV microvessels (S7C Fig) with important roles in hemostasis and vascular infiltration [61]. The pericyte (SMC3) population identified in this study (defined by *STEAP4*, *APOE*, *CD36*, *EDNRB*, *CYGB*) is most closely related to the SMC2 cluster as indicated by pseudotime trajectory analysis (S6A and S6B Fig). From the transcriptional regulation standpoint, pericytes share expression of *SRF*, *MRTFA*, and *MRTFB* with the rest of the SMCs but lack *MYOCD*. The former also show upregulation of *NOTCH3*, *PPARG* and *TBX2* similar to SMC2 cells, but significantly higher levels of *NR2F2* compared to both SMC populations (S6B Fig, **S2 File in** S1 Data). Unique genes expressed in pericytes highlight the contribution of these cells to vascular maintenance, hemostasis, and inflammation. Upregulation of endothelin receptor type B (*EDNRB*) and cytoglobin (*CYGB*) supports their role in regulating microvessel diameter and oxygen diffusion. The combination of *CD36*, *APOE*, *STEAP4*, and *FABP4* indicates a cellular phenotype primed for lipid uptake and metabolism; whereas *THY1* is a cell surface ligand that supports leukocyte infiltration (S6A Fig, **S2 File in** S1 Data) [62].

### Fibroblasts as regulators of EC survival and SMC proliferation

**Fig 4:**
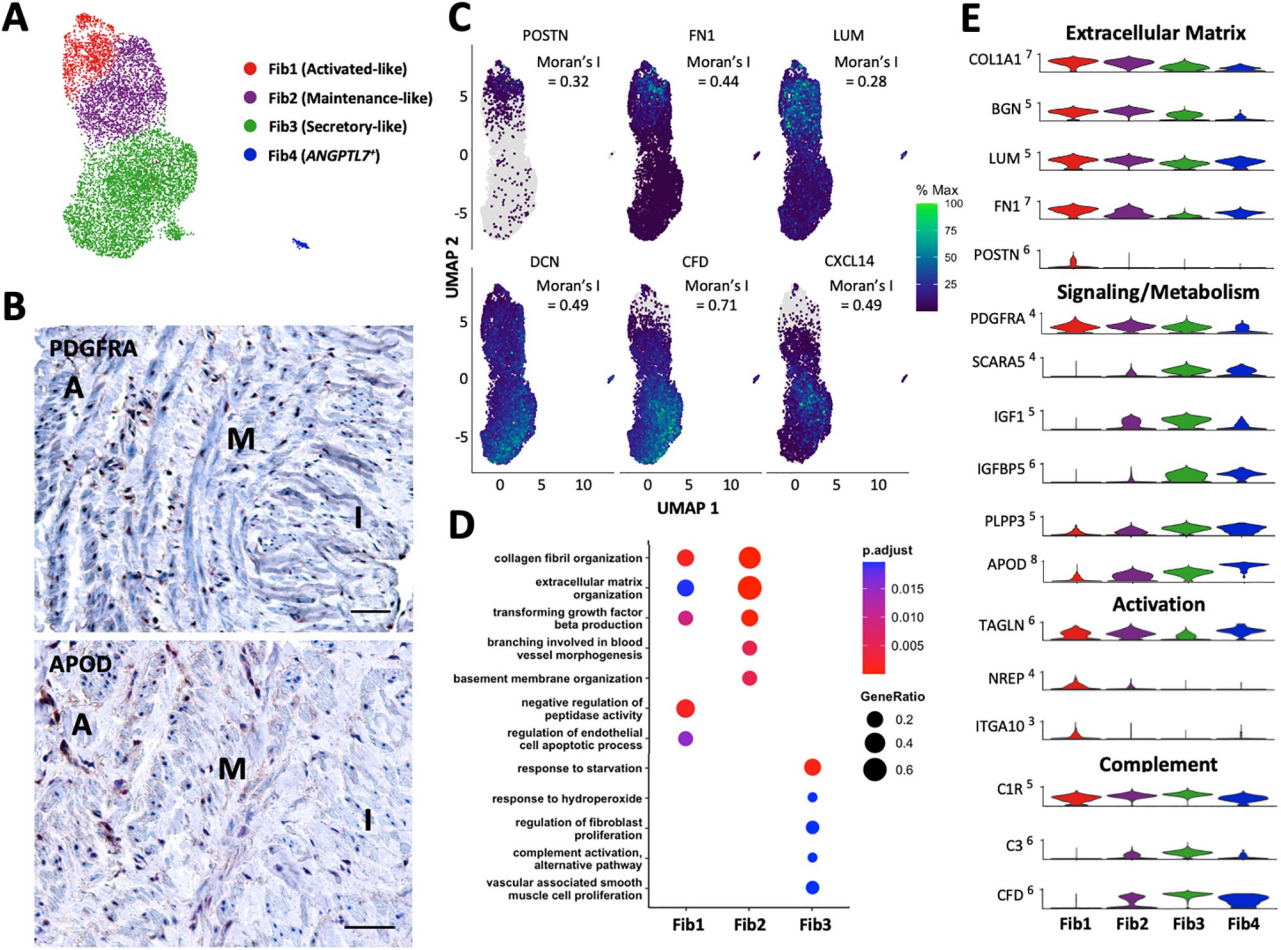
Fibroblasts in upper arm veins. **A)** Focused UMAP of 9,172 fibroblasts isolated from upper arm veins. **B)** Localization of PDGFRA^+^ (pan-fibroblast marker) and APOD^+^ (secretory marker) fibroblasts by immunohistochemistry. Scale bars represent 50 μm. **C)** Feature plots indicating the relative expressions of fibroblast markers across a pseudotime defined by transcriptional similarities among cells. The Moran’s I statistics of spatial autocorrelation is shown for each gene. **D)** Gene ontology pathways unique and in common among the 3 main fibroblast subtypes. **E)** Violin plot representation of expression differences among fibroblast populations.

The proportions of fibroblasts ranged from 14.0 to 46.9% across the 4 veins analyzed (Fig 1C), for a total of 9,172 cells out of 20,006 in the focused UMAP (Fig 4A), and more than 4X the SMC count in these tissues. Immunohistochemistry (IHC) analyses of PDGFRA, a marker in common for all venous fibroblasts, underscored their abundance and widespread distribution in the wall. Specifically, they contribute to the population of intimal cells; they are found interspersed among SMC bundles in the media, and among elastic fibers in the adventitia (Fig 4B, S8A Fig). Their presence in the intima and media also suggests an important contribution to chronic wall remodeling, even in the absence of vascular injury. Venous fibroblasts subdivided into three main subclusters (activated, maintenance, and secretory), named according to their expression of myofibroblast markers, core ECM genes, and complement/adipogenic factors, respectively. Separation into these subsets was mainly done to facilitate their phenotypic characterization (Table 1), but in reality, they organized following opposing gradients of ECM gene expression and secretory factor levels (Moran’s I > 0.28, padj<0.001) as demonstrated by pseudotime trajectory analyses (Fig 4C).

Both activated (1,637 cells) and maintenance fibroblasts (2,073 cells) showed upregulation of collagens, proteoglycans, and other ECM proteins and regulators (*COL1A1*, *COL1A2*, *COL3A1*, *BGN*, *TNC*, *LUM*, *TIMP1*) compared to the secretory subset (Fig 4C–4E). They also overexpressed the adipogenic receptor *CDH13*, the vasoconstrictive receptor *CMKLR1*, the thrombin receptor *F2R*, and as in the case of SMC2 cells, *NDUFA4L2*, a component of the mitochondrial electron transport chain associated with adaptation to hypoxic conditions (**S4 File in** S1 Data). In addition, expression of *DKK3* and *MICAL2* suggests a baseline propensity for myofibroblastic activation as a result of additional cellular or mechanical stressors [63, 64]. This phenotypic transition was already observed in the activated fibroblast cluster, which were transcriptionally related to SMC1 cells by pseudotime analysis (S8C Fig). It is also possible that activated fibroblasts represent a synthetic SMC population. The former display significant upregulation of *ACTA2*, *TAGLN*, *FN1*, the collagen-binding integrin subunit *ITGA10*, the co-activator of TGFβ translation *NREP* [65], and exclusive expression of periostin (*POSTN*) with respect to other fibroblasts (Fig 4E, **S4 File in** S1 Data). In terms of TFs, activated and maintenance fibroblasts share expression of *NOTCH3* with SMCs and pericytes, and of *FOXC2* with the latter populations and the valvular and lymphatic EC subclusters.

The most numerous fibroblast population, termed secretory fibroblasts (5,462 cells), was characterized by upregulation of complement factors (*C1S*, *C1R*, *C3*, *CFD*), angiogenesis regulators (*CD34*, *CXCL14*, *SFRP1*, *SVEP1*), and hemostatic proteins (*ACKR3*, *PTGDS*) compared to the above fibroblast subsets (Fig 4E, **S4 File in** S1 Data). High expression of genes related to adipocytic differentiation (*SCARA5*), lipid metabolism (*IGF1*, *IGFBP5*, *PLA2G2A*, *PLPP3*), and particularly, reduction of lipid peroxidation (*GPX3*, *MGST1*), suggests a role of secretory fibroblasts in lipid scavenging and detoxification. While they do not have high expression of ECM proteins, they do express genes that promote fibrotic remodeling (*CILP*, *PI16*, *CXCL14*). This secretory population, characterized by high *APOD* expression, was also widely distributed throughout the wall (Fig 4B, S8B Fig). Lastly, a small *ANGPTL7*^+^ fibroblast population (284 cells; Fig 4A) with potential pro-fibrotic effects [66] was detected in agreement with previous studies [6].

**Fig 5:**
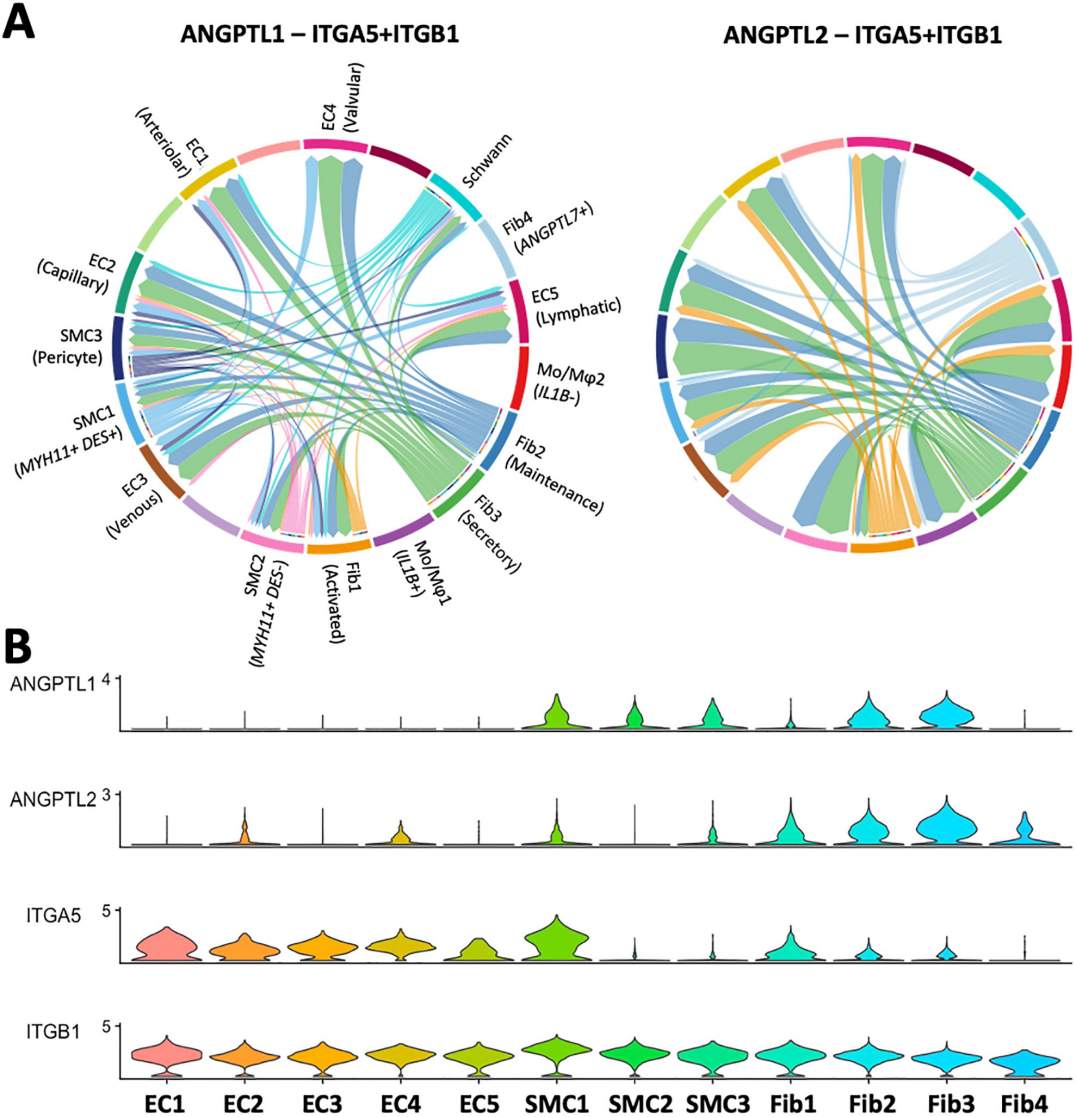
Regulation of ECs and SMCs by fibroblasts. **A)** Angiopoietin-like factors 1 and 2 secreted by maintenance, secretory, and *ANGPTL7*^+^ fibroblasts regulate ITGA5-expressing ECs and SMC1 as predicted by CellChat ligand-receptor interactome analysis. Arrows inside bubbles indicate the direction of regulation, with the size of the incoming arrow representing the strength of the interaction. **B)** Expression levels of *ANGPTL1*, *ANGPTL2*, and the alpha (*ITGA5*) and beta (*ITGB1*) subunits of the integrin receptor in ECs, SMCs, and fibroblasts.

All three fibroblast phenotypes modulate EC and SMC function through cell-to-cell interactions. Specifically, angiopoietin-like factors (*ANGPTL1*, *ANGPTL2*) produced at different levels by the three fibroblasts are predicted to interact with ITGA5 expressing cells, to regulate EC survival, vascular permeability, promote proliferation and migration of medial SMCs, and increase immune cell infiltration (Figs 4D and 5) [66–69]. Upregulation of *IGFBP5* in SMCs is also expected to potentiate the effects of fibroblast-derived *IGF1* in inducing SMC proliferation (Fig 4D, S8D Fig) [70].

### The venous endothelium is a niche for monocyte recruitment and infiltration

**Fig 6:**
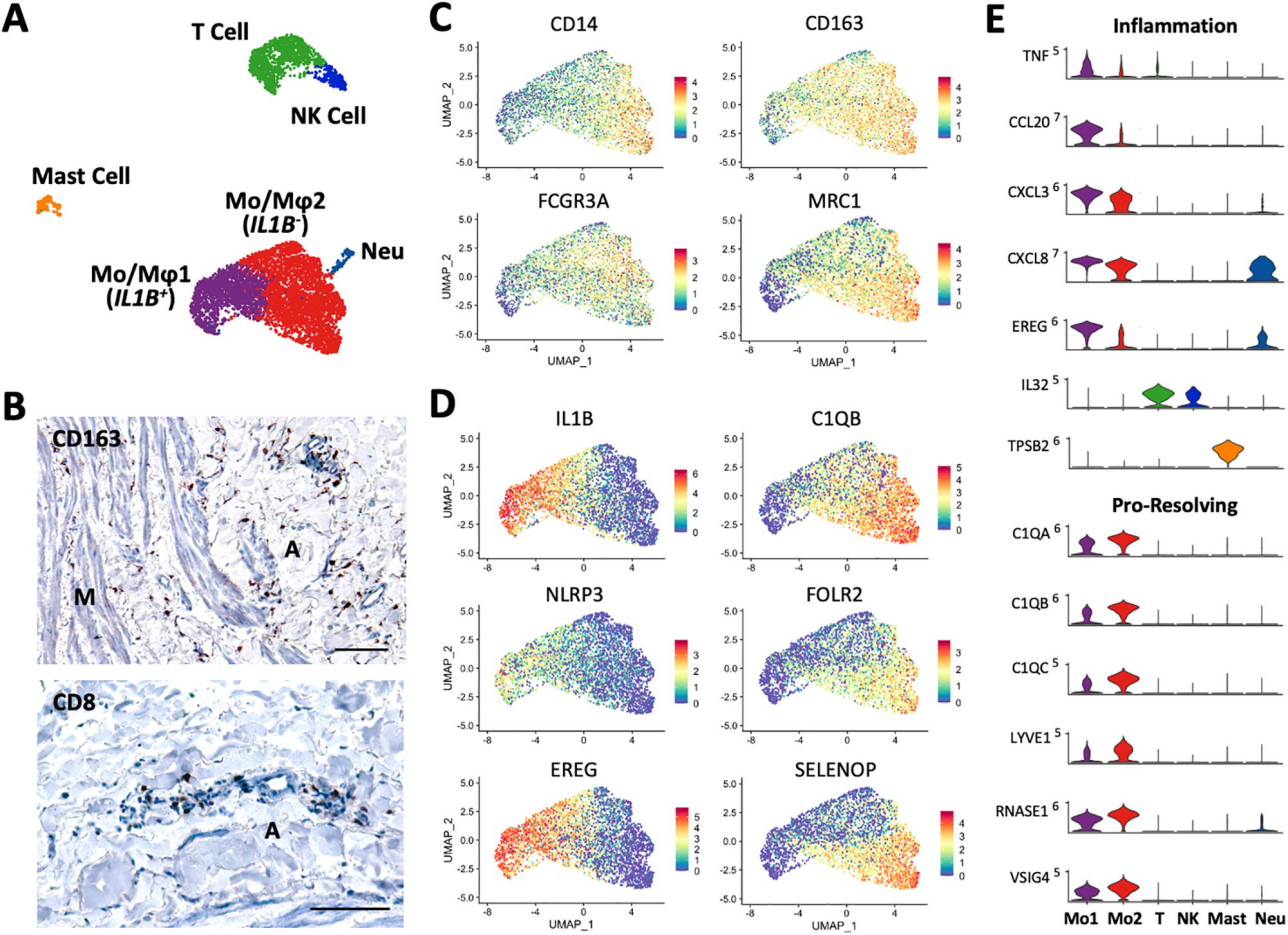
Immune cells in upper arm veins. **A)** Focused UMAP of 4931 monocytes/macrophages (Mo/Mφ), 1165 T cells, 244 NK cells, 235 mast cells, and 142 neutrophils (Neu) isolated from upper arm veins. **B)** Localization of CD163^+^ monocytes/macrophages and CD8^+^ T cells by immunohistochemistry. Scale bars represent 100 μm. **C-D)** Feature plots indicating normalized expression level of markers in common (C) and different (D) between the two monocyte/macrophage subpopulations. **E)** Violin plot representation of expression differences among immune cell populations.

One of the most intriguing findings of our study is the high abundance of immune cells in healthy upper arm veins (Fig 1B and 1C, S9 Fig). There are a total of six immune subpopulations in the 4 veins analyzed, including 4931 monocytes/macrophages, 1165 T cells, 244 NK cells, 235 mast cells, and 142 neutrophils (Figs 1C, 6A and 6B). Monocytes/macrophages comprise the most prominent immune cluster, accounting for 18.3 to 43.8% of cells in samples. Counts of CD163^+^ monocyte/macrophages positively correlate with donor’s age in histological sections (S9D Fig), which may explain the higher proportion of this cell cluster in samples BV1 and BV3 (Fig 1C). NK cells (0.5–2.5%), mast cells (0.4–1.3%), and neutrophils (0.3–1.9%) are found in smaller proportions. T lymphocytes (29.6% *CD8A*^+^, 7.6% *CD4*^+^) are present at low proportions in three of the veins (1.3–5.8%), but at 25.6% in the fourth one. Unlike monocyte/macrophages, there is no correlation between CD8^+^ cell counts by histology and donor’s age (S9D Fig).

The overall monocyte/macrophage cluster can be identified by expression of CD14, CD16 (*FCGR3A*), CD163, and CD206 (*MRC1*) (Fig 6C). These further subdivided into two subclusters named according to other transcriptional features: Mo/Mφ1 (2,012 cells), which show upregulation of *IL1B*, *CCL20*, *CXCL3*, *CXCL8*, *TNF*, *EREG*, and the inflammasome sensor *NLRP3*; and Mo/Mφ2 (2,919 cells), a reparative phenotype that has been previously reported in non-vascular tissues [71–73]. The latter are characterized by high expression of *C1QA/B/C*, *FOLR2*, *HMOX1*, *LYVE1*, *RNASE1*, *SELENOP*, and *VSIG4* (Fig 6D and 6E, **S5 File in** S1 Data). Along with CD163, this combination of genes is associated with hemoglobin and heme scavenging, apoptotic cell clearance (efferocytosis), and resolution of inflammation [74–76].

**Fig 7:**
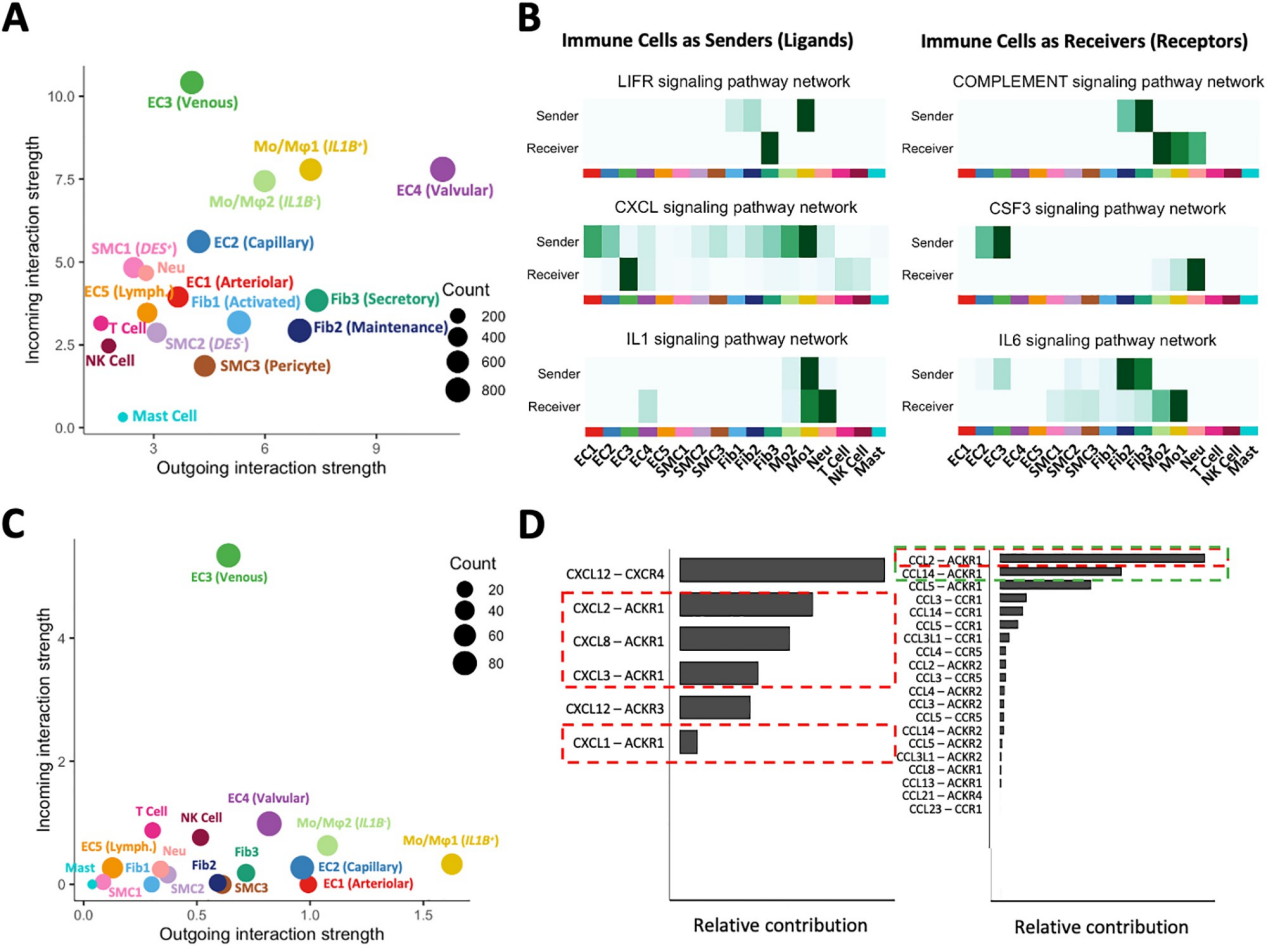
Ligand-receptor interactions among venous cell populations. **A)** Global plot of outgoing (ligand) and incoming (receptor) interactions among cell populations in veins as predicted by CellChat. Venous ECs are the top receivers among all populations. Valvular ECs are the top senders of signals. Monocytes/macrophages (Mo/Mφ) are the main immune populations participating as senders and receivers of global interactions. **B)** Examples of pathways where immune cells act as senders (left) or receivers (right) of signals. **C)** Plot of outgoing and incoming interactions among cell populations specifically for the chemokine (CXCL/CCL) signaling pathway. Venous ECs are the main receivers of signals through the ACKR1 receptor, while *IL1B*^+^ Mo/Mφ are the top senders. **D)** Relative contribution of specific chemokines to the plot in C. Red boxes indicate chemokines secreted by *IL1B*^+^ Mo/Mφ, while the green box marks chemokines secreted by venous/capillary ECs (CCL14) or other cells including venous ECs (CCL2).

Both monocyte/macrophage phenotypes were the most important immune cells contributing to outgoing (ligand secretion) and incoming (receptor binding) interactions with other cells in the vascular wall (Fig 7A and 7B). Furthermore, GO analyses identified the pro-inflammatory Mo/Mφ1 population as the main immune cell type affecting survival, proliferation, and differentiation of ECs and SMCs (S9A Fig). Specifically, Mo/Mφ1-derived *IL1B* and *TNF* upregulate adhesion molecules, pro-inflammatory mediators, and pro-coagulant factors in ECs (Fig 7B, S9A Fig) [77–79]. Thrombospondin-1 (*THBS1*) produced by pro-inflammatory Mo/Mφ1 cells has anti-proliferative properties for ECs but positively regulates SMC proliferation and migration [80, 81]. Mo/Mφ1-derived epiregulin (*EREG*) is also a potent SMC mitogen (S9A Fig, **S5 File in** S1 Data) [82]. In addition to ECs and SMCs, Mo/Mφ1 cells promote survival of secretory fibroblasts through secretion of *CLCF1* and activation of the fibroblast *CNTFR* receptor (LIFR pathway in Fig 7B). Fibroblasts, in turn, are important regulators of macrophage function via secretion of complement factors and IL6. Both Mo/Mφ1 cells and venous ECs activate and increase survival of infiltrated neutrophils through production of *IL1* factors and *CSF3*, respectively (Fig 7B).

Venous ECs are specifically affected by macrophage-derived chemokines *CXCL1*, *2*, *3*, and *8* via activation of the ACKR1 receptor (Fig 7B–7D). Additional chemokines modulating venous ECs include *CCL2* from all cells except T and NK cells, *CCL14* from other venous and capillary-like ECs, and *CCL5* from T lymphocytes. Of note, regardless of the cellular origin of these cytokines and chemokines, the ability of ACKR1 to allow transcytosis suggests that the main lumen of veins and VV venules maintain a microenvironment that serves as beacon for monocytes. Immunohistochemistry analyses demonstrate that CD163^+^ monocytes/macrophages and CD8^+^ T cells are abundant in the adventitia, particularly in the vicinity and lumen of microvessels (Fig 6B, S9B and S9C Fig).

## Discussion

Peripheral veins are the preferred anatomical location for creation of vascular accesses for hemodialysis (arm veins) and common conduits for coronary bypass surgeries (saphenous vein). This single-cell transcriptomic study demonstrates for the first time that: 1) veins harbor a cellular ecosystem of abundant fibroblasts and immune cells in much higher proportions than SMCs; 2) the existence of VV consisting of a complex populaton of ECs of various arteriovenous zonation phenotypes; 3) a unique transcriptional profile in venous SMCs; and 4) the widespread presence of regulatory, reparative, and interacting fibroblasts in all layers of the vascular wall. This granular knowledge of the cellular composition and transcriptional states of cells in human peripheral veins may lead to a better understanding of venous physiology and mechanisms underlying diseases and post-operative complications.

The heterogeneity of EC phenotypes as defined by arteriovenous zonation markers illustrates at the transcriptional level the ECs lining the intima, and the complex microvascular structures of VV irrigating the outer media and adventitia. The venous VV has been previously described with elegant 3D renderings of scanning electron microscopy [24], but little is known about this vascular network at the molecular level. The majority of ECs were of the venous type that histological located in the endothelial line of the lumen and the extensive network of draining venous VV [24], We also identified an abundant capillary EC population that went unnoticed in prior studies [25], and which may be important for expansion of the VV during postoperative remodeling or, in the case of lower extremities, for the development of chronic venous insufficiency. The high expression of adhesion molecules in venous ECs, upregulation of pro-inflammatory mediators, and ACKR1-mediated transcytosis of chemokines [83] redefine the concept of endothelial activation, and underscores the role of the main venous lumen and VV venules as preferential sites of infiltration and a niche for both reparative and pro-inflammatory immune cell populations.

We also demonstrated that SMCs in human veins are positive for *MYH11* and other markers of contractility, but have additional transcriptional features that challenge this arterial-based phenotypic classification. Synthetic SMCs in single-cell studies of arteries have been identified as having low expression of contractile genes such as *MYH11*, *ACTG*, and *CNN1* and high levels of ECM genes [1, 3, 10]. In contrast, two transcriptional states of *MYH11*^+^ SMCs coinhabited the intima and media in veins, distinguished by upregulation of *DES* and *ITGA5* in one state and of RhoA signaling proteins in the other. Upregulation of *ITGA5*, fibronectin, and collagens by *DES*^*hi*^ SMCs supports an active ECM remodeling phenotype and high migratory and proliferative potential [48, 49]. On the other hand, *DES*^*lo*^ SMCs express genes associated with negative regulation of contractility and have higher pro-angiogenic and -inflammatory gene expression. Historically, the “contractile” phenotype has been defined by high expression of genes in the actomyosin apparatus (particularly *MYH11*) and location in the media, but not necessarily by functional assessment. Considering that the main function of veins is not contractility, that arms veins specifically have a negligible myogenic tone [84], and that contractile proteins also play crucial functions in cell migration [85], this “contractile” phenotype in veins calls for future investigations. Along these lines, the value of desmin as a contractile marker in bladder SMCs has been recently debated [86].

Stenotic remodeling and IH in veins are often associated with expansion of a synthetic SMC phenotype and migration of activated fibroblasts from the adventitia after vascular injury [16, 87]. Interestingly, we did not observe a clear transcriptomic distinction between medial and intimal SMCs, or between spindle-shaped or rounded SMC morphologies in these layers. Furthermore, there was a widespread distribution of fibroblasts in the walls of these veins, interspersed among SMCs in the intima and media. It is likely that in the absence of well formed internal and external elastic laminae, the structure of the vein is fully permissive to cell migration. Therefore, unlike arterial diseases, the cells contributing to venous IH may not need to dedifferentiate extensively or migrate from the adventitia. Phenotypic similarities between intimal and medial SMCs, their pro-remodeling transcriptional profiles, and the widespread distribution of fibroblasts may also explain the tendency of veins to chronically develop IH and fibrosis [15]. Ultimately, comparative studies with veins obtained after vascular surgeries will be necessary to evaluate the true cellular contributors to postoperative IH and their molecular differentiation.

Lastly, cell-to-cell signaling underscored the versatility of venous fibroblasts in regulating immune cell populations, ECs, and SMCs through complement factors, pro-inflammatory mediators, and angiopoietin-like proteins. Like ECs, venous fibroblast phenotypes may contribute to perpetuating or resolving vascular inflammation. Secretory fibroblasts are also important regulators of intramural vascularization [88].

In conclusion, this study presents for the first time, to our knowledge, a detailed cellular atlas of upper arm veins with molecular information that is particularly relevant to the study of post-surgical venous remodeling after vascular access creation. Our data provide the groundwork for future molecular studies and single-cell characterizations of veins in the setting of vascular injury and patient comorbidities. We present compelling evidence of the venous endothelium as a preferential site of immune surveillance and infiltration, and of complex SMC/non-SMC interactions in the intima and media that may underlie clinical outcomes after vascular procedures.

## Materials and methods

### Sample collection and tissue processing

The study cohort included 16 organ donors whose tissues were donated for research through a collaboration with the Life Alliance Organ Recovery Agency (no informed consent required; S1 Table). Cross-sectional samples of upper arm vessels (12 basilic veins, 3 cephalic veins, 1 brachial artery), approximately 2 cm in length, were obtained *post mortem* following organ procurement procedures. For single-cell RNA sequencing, 4 veins (3 basilic, 1 cephalic) were collected in cold cell culture media cold for tissue dissociation (S1 File). The remaining 12 samples were collected in RNA*later* (QIAGEN, Germantown, MD) and stored at -80°C. A 5 mm cross-section was fixed in 10% neutral formalin (Sigma-Aldrich, St. Louis, MO) for paraffin embedding and sectioning.

### Single-cell RNA sequencing and bioinformatic analyses

Single-cell RNA sequencing was performed in the Center for Genome Technology at the University of Miami John P. Hussman Institute for Human Genomics. Sequencing data were processed in R (4.2.2) using Seurat v4 and available packages for quality control (QC), integration, and bioinformatic inferences [89–95] as described in the S1 File. Briefly, a total of 31,109 cells were obtained after 10X droplet generation and single cell sequencing. For quality control (QC), we adjusted for ambient RNA contamination and filtered out predicted doublets and cells with >15% mitochondrial genes and <200 genes, resulting in 21,131 high quality cells for downstream analysis (S2A and S2B Fig). We then excluded clusters with less than 40 cells and those defined by markers of cell division (*CENPF*, *MKI67*, *TOP2A*; 697 cells). The integrated map was generated using the package Harmony [92]. Sequencing data have been deposited in the Gene Expression Omnibus (GEO), accession number GSE250469.

### Histology and immunohistochemistry analyses

Tissue sections were stained with Masson’s (Polysciences #25088–1) trichrome and Movat’s pentachrome (Abcam #ab245884) for gross histomorphometric analysis. For IHC and IF, specific proteins were detected using the primary antibodies and methodology described in the S1 File.

## Supporting information

S1 Table Demographic characteristics of the single-cell RNA sequencing and histological validation cohorts. (PDF)

S1 Fig Histology of upper arm vessels. Representative Movat pentachrome stained sections of vascular tissues used for validation studies. Images include three basilic veins (**A**), two cephalic veins (**B**) and a brachial artery as reference (**C**). Dashed boxes are magnified to the right.
(PDF)

S2 Fig Quality control (QC) metrics of single-cell RNA libraries. **A)** Number of genes versus number of UMIs (transcripts) per cell after initial QC filters with color legend indicating mitochondrial read fractions (mitoRatio). **B)** Cell density histograms indicating distribution of UMI counts and gene counts per cell in the four vein samples. Cells with <200 genes, >0.15 mitoRatio, or predicted as doublets were filtered out from downstream bioinformatic analyses. **C)** UMAP plot color coded by sample demonstrating cell integration of individual single cell libraries.
(PDF)

S3 Fig Transcriptional profiling of endothelial cell (EC) populations in veins. **A)** Heatmap of single-cell gene expression data for the top differentially expressed genes among EC subclusters in veins. Genes are shown in rows and cells in columns, color-coded according to the main arteriovenous zonation phenotypes (EC1: arteriolar-like, EC2: capillary-like, EC3: venous-like, EC4: valvular-like, and EC5: lymphatic-like). **B)** Feature plots indicating the relative expressions of transcription factors and ephrinB2 ligand and receptor markers across a pseudotime defined by arteriovenous zonation phenotypes. The pseudotime direction goes from EC1 to EC4 followed by EC5, with the Moran’s I statistics of spatial autocorrelation shown for each gene. **C)** Gene expression levels of positive (*SNAI1*) and negative regulators (*EPB41L3*) of endothelial to mesenchymal transition in the five EC subclusters.
(PDF)

S4 Fig Venous endothelial cells in the lumen and vasa vasorum. **A)** Representative co-immunofluorescence of the venous EC markers PLVAP and ACKR1 in the main lumen (left) and venous vasa vasorum (right). The yellow color indicates colocalization of both markers in the lumen and venules. **B)** Co-immunofluorescence of PLVAP and GJA5 in the main lumen (left) and vasa vasorum (right). There is low protein expression of GJA5 in the lumen and venules, while localization of GJA5 and PLVAP discriminates between arterioles (Art.) and venules (Ven.) in the vasa vasorum. Scale bars represent 20 μm in all panels.
(PDF)

S5 Fig Predicted functional differences among cell populations in veins. Gene ontology pathway analysis of genes expressed in endothelial cell (**A**) and smooth muscle cell (**B**) populations in veins. EC1: arteriolar-like, EC2: capillary-like, EC3: venous-like, EC4: valvular-like, EC5: lymphatic-like, SMC1: *MYH11*^+^*DES*^*hi*^, SMC2: *MYH11*^+^*DES*^*lo*^, SMC3: pericyte-like.
(PDF)

S6 Fig Transcriptional profiling of smooth muscle cell (SMC) populations in veins. **A)** Heatmap of single-cell gene expression data for the top differentially expressed genes among SMC subclusters in veins. Genes are shown in rows and cells in columns, color-coded by subcluster (SMC1: *MYH11*^+^*DES*^*hi*^, SMC2: *MYH11*^+^*DES*^*lo*^, and SMC3: pericyte-like). **B)** Feature plots indicating the relative expressions of transcription factors across a pseudotime defined by transcriptional similarities among cells. The pseudotime direction goes from SMC1 to SMC3, with the Moran’s I statistics of spatial autocorrelation shown for each gene. **C)** The SMC2 population is the main source of placental growth factor (*PGF*) in the vein with pro-angiogenic effects on *FLT1*-expressing EC1, EC2, EC3, and pro-inflammatory monocyte/macrophages, as predicted by CellChat ligand-receptor interactome analysis. Arrows in the bubble indicate the direction of regulation, with the size of the incoming arrow representing the strength of the interaction.
(PDF)

S7 Fig Types of smooth muscle cells in veins. **A)** Representative co-immunofluorescence of MYH11 and desmin in medial (left) and intimal SMCs (right). Cells with high (yellow-orange) and low desmin expression (green) coexist in both layers. **B)** Co-immunofluorescence of MYH11 and ITGA5 in medial (left) and intimal SMCs (right) shows similar distribution of ITGA5 high and low cells as in **A**. **C)** STEAP4+ pericytes surrounding microvessels. Scale bar = 20 μm in all panels.
(PDF)

S8 Fig Venous fibroblasts. **A)** Representative IHC of PDGFRA^+^ fibroblasts in veins demonstrates widespread distribution in the wall. Scale bars = 50 μm. **B)** Immunofluorescence of APOD in the intima (I), media (M), and adventitia (A). APOD^+^ fibroblasts appear in pink while extracellular APOD is shown in red. Scale bars = 20 μm. **C)** The Fib1 cluster is most closely related to SMC1 cells as predicted by pseudotime trajectory analysis. **D)** *IGFBP5* in SMCs can potentiate the effects of insulin-like growth factor 1 (*IGF1*) from fibroblasts to promote SMC proliferation.
(PDF)

S9 Fig Immune cells in veins. **A)** Gene ontology pathway analysis of genes expressed in immune cell populations in veins. Mo: monocyte/macrophages, NK: natural killer cells, Mast: mast cells, Neu: neutrophils. **B-C)** Representative stainings of CD163^+^ monocyte/macrophages (**B**) and CD8^+^ T cells (**C**) in basilic veins from the validation cohort. Scale bars represent 50 μm in all panels. A: adventitia, M: media. D) Donor’s age is positively correlated with CD163^+^ cell counts, but not CD8^+^ counts, in 6–7 basilic veins analyzed by IHC.
(PDF)

S1 File Detailed methodology of sample collection, tissue processing, single-cell RNA sequencing, bioinformatic pipelines, and histological validations. (PDF)

S1 Data Cell population markers. Cell markers and gene expression differences among endothelial cell (S1), smooth muscle cell (S2-S3), fibroblast (S4), and immune cell (S5) subpopulations as determined by Wilcoxon signed ranks tests.
(XLSX)
