# Supplementary material for: The intricate cellular ecosystem of human peripheral veins as revealed by single-cell transcriptomic analysis
Source: PLoS One. 2024 Jan 11;19(1):e0296264. doi: 10.1371/journal.pone.0296264 (PMC10783777; doi:10.1371/journal.pone.0296264)
Supplement: S1 Table — (PDF) [file pone.0296264.s001.pdf]

S1 Table. Demographic characteristics of the single-cell RNA sequencing and histological validation cohorts

Sample ID	Age	Sex	Race/Ethnicity	Blood Vessel	# Cells After QC*
**Single-Cell RNA Sequencing Cohort**					
CV1	26	F	Black	Cephalic V.	4,663
BV1	70	M	White	Basilic V.	3,473
BV2	38	F	Hispanic	Basilic V.	10,819
BV3	64	M	White	Basilic V.	1,051
**Histological Validation Cohort**					
CV2	56	M	Hispanic	Cephalic V.	NA
CV3	55	M	Hispanic	Cephalic V.	NA
BV4	65	M	White	Basilic V.	NA
BV5	30	M	White	Basilic V.	NA
BV6	56	M	Hispanic	Basilic V.	NA
BV7	45	F	White	Basilic V.	NA
BV8	24	F	White	Basilic V.	NA
BV9	52	M	White	Basilic V.	NA
BV10	21	M	White	Basilic V.	NA
BV11	41	M	White	Basilic V.	NA
BV12	29	M	Hispanic	Basilic V.	NA
BA1	56	M	White	Brachial A.	NA

*****_Number of cells included in bioinformatic analyses after quality control (QC) filters_

Abbreviations: V, vein; A, artery; NA, not applicable
