# Supplementary material for: The intricate cellular ecosystem of human peripheral veins as revealed by single-cell transcriptomic analysis
Source: PLoS One. 2024 Jan 11;19(1):e0296264. doi: 10.1371/journal.pone.0296264 (PMC10783777; doi:10.1371/journal.pone.0296264)
Supplement: S1 Fig — Representative Movat pentachrome stained sections of vascular tissues used for validation studies. Images include three basilic veins (A), two cephalic veins (B) and a brachial artery as reference (C). Dashed boxes are magnified to the right. (PDF) [file pone.0296264.s002.pdf]

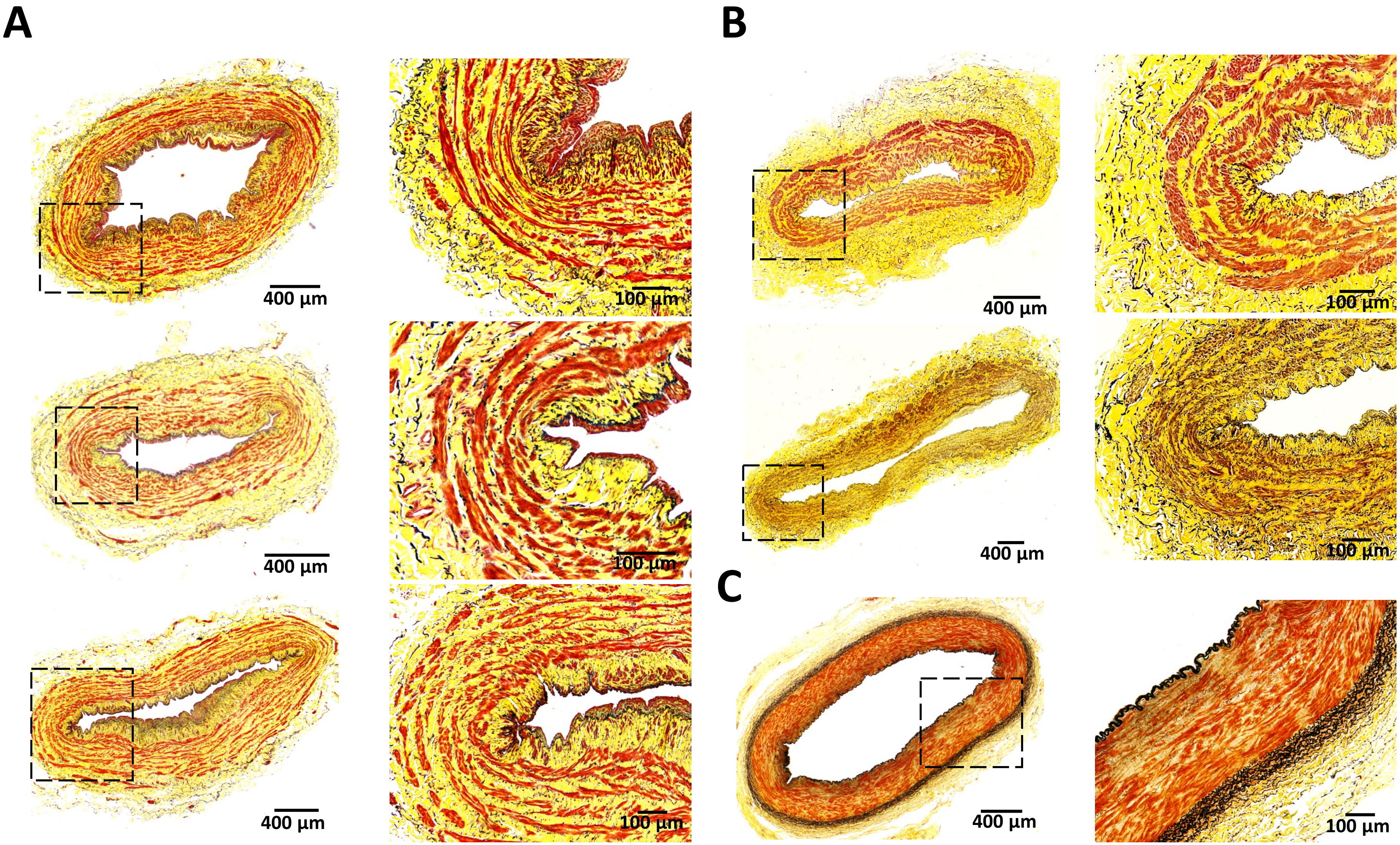

S1 Fig. Histology of upper arm vessels. Representative Movat pentachrome stained sections of vascular tissues used for validation studies. Images include three basilic veins (**A**), two cephalic veins (**B**), and a brachial artery as reference (**C**). Dashed boxes are magnified to the right.
