# Supplementary material for: The intricate cellular ecosystem of human peripheral veins as revealed by single-cell transcriptomic analysis
Source: PLoS One. 2024 Jan 11;19(1):e0296264. doi: 10.1371/journal.pone.0296264 (PMC10783777; doi:10.1371/journal.pone.0296264)
Supplement: S2 Fig — A) Number of genes versus number of UMIs (transcripts) per cell after initial QC filters with color legend indicating mitochondrial read fractions (mitoRatio). B) Cell density histograms indicating distribution of UMI counts and gene counts per cell in the four vein samples. Cells with <200 genes, >0.15 mitoRatio, or predicted as doublets were filtered out from downstream bioinformatic analyses. C) UMAP plot color coded by sample demonstrating cell integration of individual single cell libraries. (PDF) [file pone.0296264.s003.pdf]

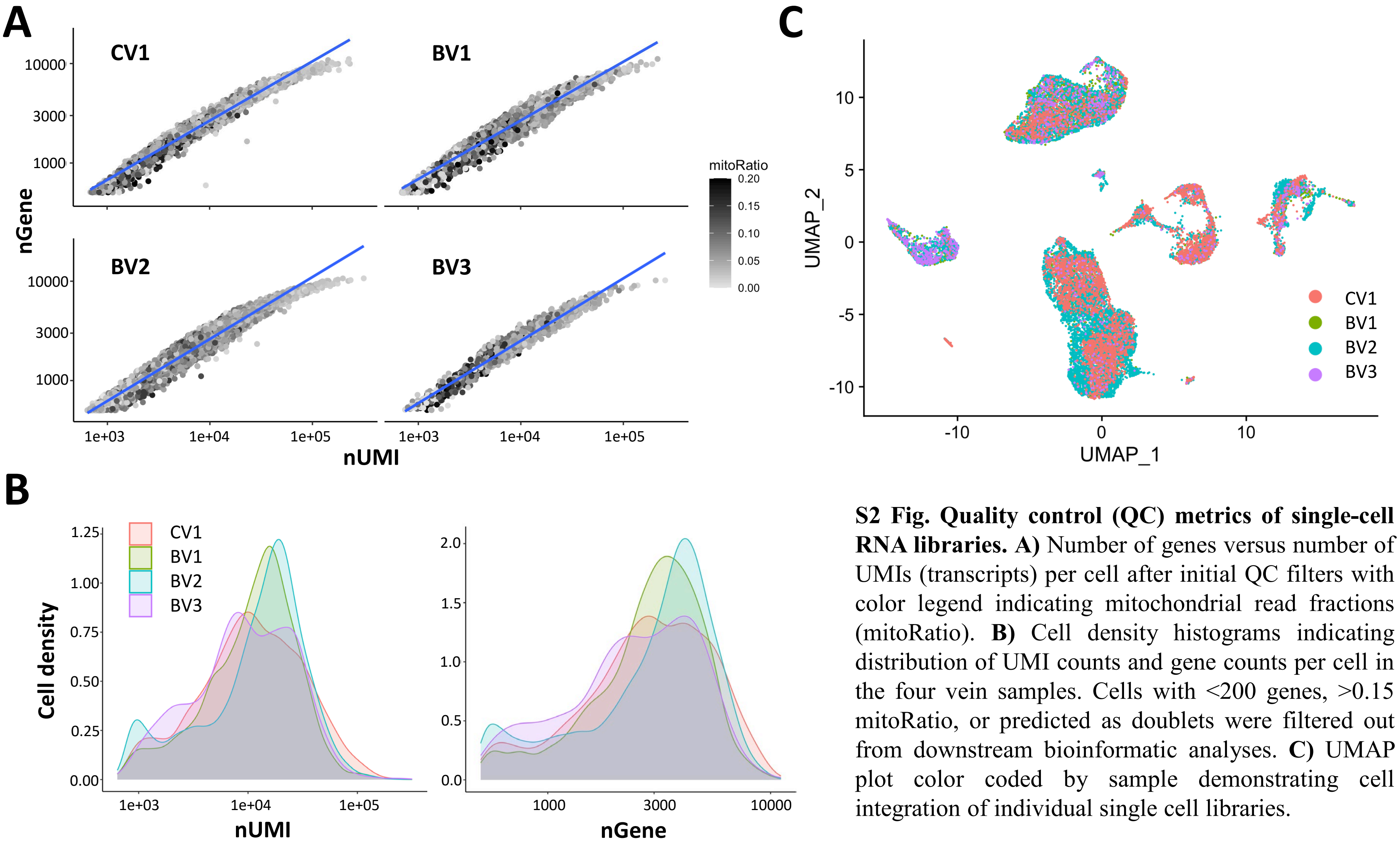


**S2 Fig. Quality control (QC) metrics of single-cell RNA libraries. A)** Number of genes versus number of UMIs (transcripts) per cell after initial QC filters with color legend indicating mitochondrial read fractions (mitoRatio). **B)** Cell density histograms indicating distribution of UMI counts and gene counts per cell in the four vein samples. Cells with <200 genes, >0.15 mitoRatio, or predicted as doublets were filtered out from downstream bioinformatic analyses. **C)** UMAP plot color coded by sample demonstrating cell integration of individual single cell libraries.
