# Supplementary material for: The intricate cellular ecosystem of human peripheral veins as revealed by single-cell transcriptomic analysis
Source: PLoS One. 2024 Jan 11;19(1):e0296264. doi: 10.1371/journal.pone.0296264 (PMC10783777; doi:10.1371/journal.pone.0296264)
Supplement: S3 Fig — A) Heatmap of single-cell gene expression data for the top differentially expressed genes among EC subclusters in veins. Genes are shown in rows and cells in columns, color-coded according to the main arteriovenous zonation phenotypes (EC1: arteriolar-like, EC2: capillary-like, EC3: venous-like, EC4: valvular-like, and EC5: lymphatic-like). B) Feature plots indicating the relative expressions of transcription factors and ephrinB2 ligand and receptor markers across a pseudotime defined by arteriovenous zonation phenotypes. The pseudotime direction goes from EC1 to EC4 followed by EC5, with the Moran’s I statistics of spatial autocorrelation shown for each gene. C) Gene expression levels of positive (SNAI1) and negative regulators (EPB41L3) of endothelial to mesenchymal transition in the five EC subclusters. (PDF) [file pone.0296264.s004.pdf]

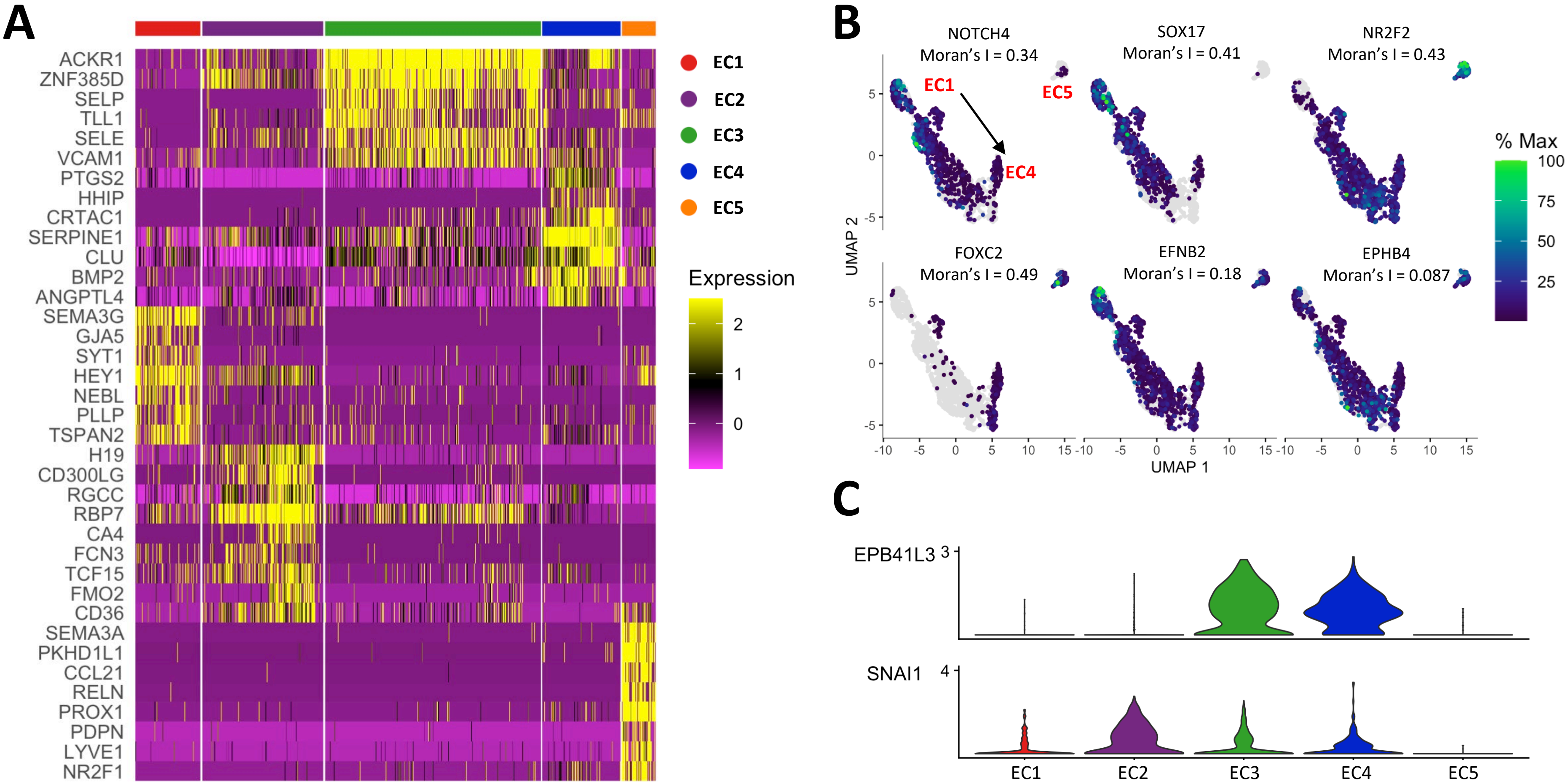

S3 Fig. Transcriptional profiling of endothelial cell (EC) populations in veins. A) Heatmap of single-cell gene expression data for the top differentially expressed genes among EC subclusters in veins. Genes are shown in rows and cells in columns, color-coded according to the main arteriovenous zonation phenotypes (EC1: arteriolar-like, EC2: capillary-like, EC3: venous-like, EC4: valvular-like, and EC5: lymphatic-like). **B)** Feature plots indicating the relative expressions of transcription factors and ephrinB2 ligand and receptor markers across a pseudotime defined by arteriovenous zonation phenotypes. The pseudotime direction goes from EC1 to EC4 followed by EC5, with the Moran's I statistics of spatial autocorrelation shown for each gene. **C)** Gene expression levels of positive (*SNAI1*) and negative regulators (*EPB41L3*) of endothelial to mesenchymal transition in the five EC subclusters.
