# Supplementary material for: The intricate cellular ecosystem of human peripheral veins as revealed by single-cell transcriptomic analysis
Source: PLoS One. 2024 Jan 11;19(1):e0296264. doi: 10.1371/journal.pone.0296264 (PMC10783777; doi:10.1371/journal.pone.0296264)
Supplement: S4 Fig — A) Representative co-immunofluorescence of the venous EC markers PLVAP and ACKR1 in the main lumen (left) and venous vasa vasorum (right). The yellow color indicates colocalization of both markers in the lumen and venules. B) Co-immunofluorescence of PLVAP and GJA5 in the main lumen (left) and vasa vasorum (right). There is low protein expression of GJA5 in the lumen and venules, while localization of GJA5 and PLVAP discriminates between arterioles (Art.) and venules (Ven.) in the vasa vasorum. Scale bars represent 20 μm in all panels. (PDF) [file pone.0296264.s005.pdf]

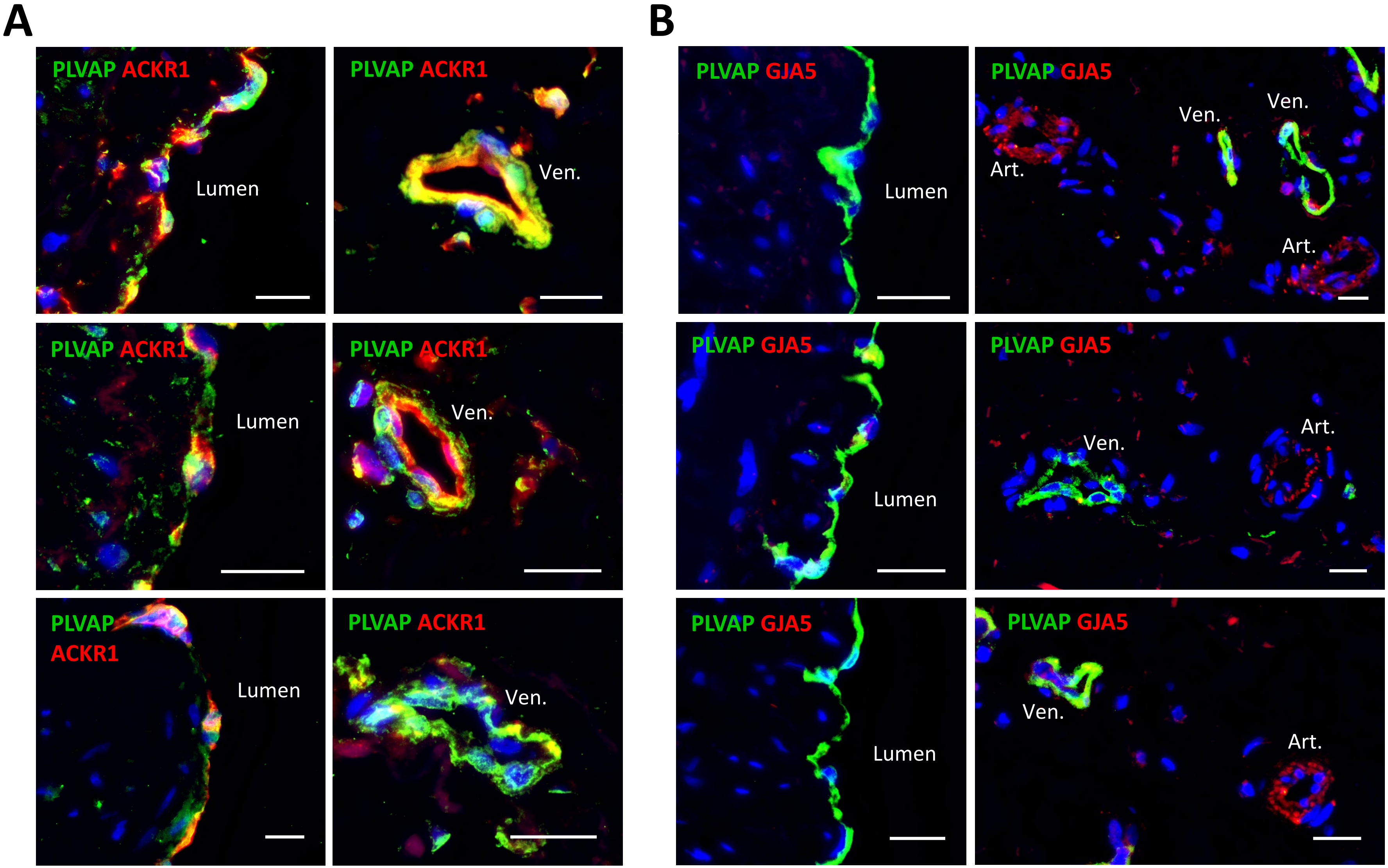

S4 Fig. Venous endothelial cells in the lumen and vasa vasorum. A) Representative co-immunofluorescence of the venous EC markers PLVAP and ACKR1 in the main lumen (left) and venous vasa vasorum (right). The yellow color indicates colocalization of both markers in the lumen and venules. **B)** Co-immunofluorescence of PLVAP and GJA5 in the main lumen (left) and vasa vasorum (right). There is low protein expression of GJA5 in the lumen and venules, while localization of GJA5 and PLVAP discriminates between arterioles (Art.) and venules (Ven.) in the vasa vasorum. Scale bars represent 20 µm in all panels.
