# Supplementary material for: The intricate cellular ecosystem of human peripheral veins as revealed by single-cell transcriptomic analysis
Source: PLoS One. 2024 Jan 11;19(1):e0296264. doi: 10.1371/journal.pone.0296264 (PMC10783777; doi:10.1371/journal.pone.0296264)
Supplement: S5 Fig — Gene ontology pathway analysis of genes expressed in endothelial cell (A) and smooth muscle cell (B) populations in veins. EC1: arteriolar-like, EC2: capillary-like, EC3: venous-like, EC4: valvular-like, EC5: lymphatic-like, SMC1: MYH11+DEShi, SMC2: MYH11+DESlo, SMC3: pericyte-like. (PDF) [file pone.0296264.s006.pdf]

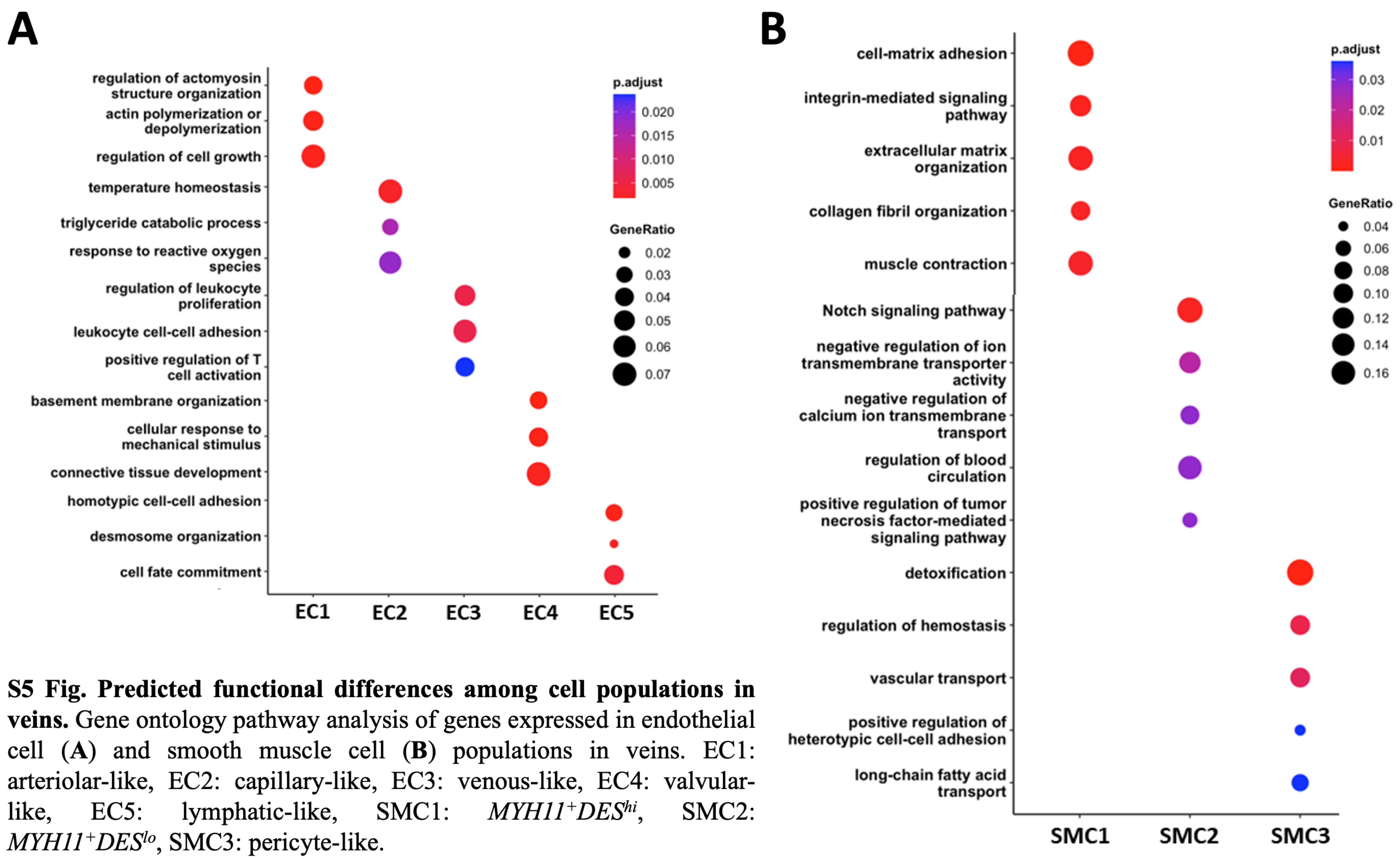

S5 Fig. Predicted functional differences among cell populations in veins. Gene ontology pathway analysis of genes expressed in endothelial cell (**A**) and smooth muscle cell (**B**) populations in veins. EC1: arteriolar-like, EC2: capillary-like, EC3: venous-like, EC4: valvular-like, EC5: lymphatic-like, SMC1: *MYH11*$^{+}$*DES*^{hi}, SMC2: *MYH11*$^{+}$*DES*^{lo}, SMC3: pericyte-like.
