# Supplementary material for: The intricate cellular ecosystem of human peripheral veins as revealed by single-cell transcriptomic analysis
Source: PLoS One. 2024 Jan 11;19(1):e0296264. doi: 10.1371/journal.pone.0296264 (PMC10783777; doi:10.1371/journal.pone.0296264)
Supplement: S6 Fig — A) Heatmap of single-cell gene expression data for the top differentially expressed genes among SMC subclusters in veins. Genes are shown in rows and cells in columns, color-coded by subcluster (SMC1: MYH11+DEShi, SMC2: MYH11+DESlo, and SMC3: pericyte-like). B) Feature plots indicating the relative expressions of transcription factors across a pseudotime defined by transcriptional similarities among cells. The pseudotime direction goes from SMC1 to SMC3, with the Moran’s I statistics of spatial autocorrelation shown for each gene. C) The SMC2 population is the main source of placental growth factor (PGF) in the vein with pro-angiogenic effects on FLT1-expressing EC1, EC2, EC3, and pro-inflammatory monocyte/macrophages, as predicted by CellChat ligand-receptor interactome analysis. Arrows in the bubble indicate the direction of regulation, with the size of the incoming arrow representing the strength of the interaction. (PDF) [file pone.0296264.s007.pdf]

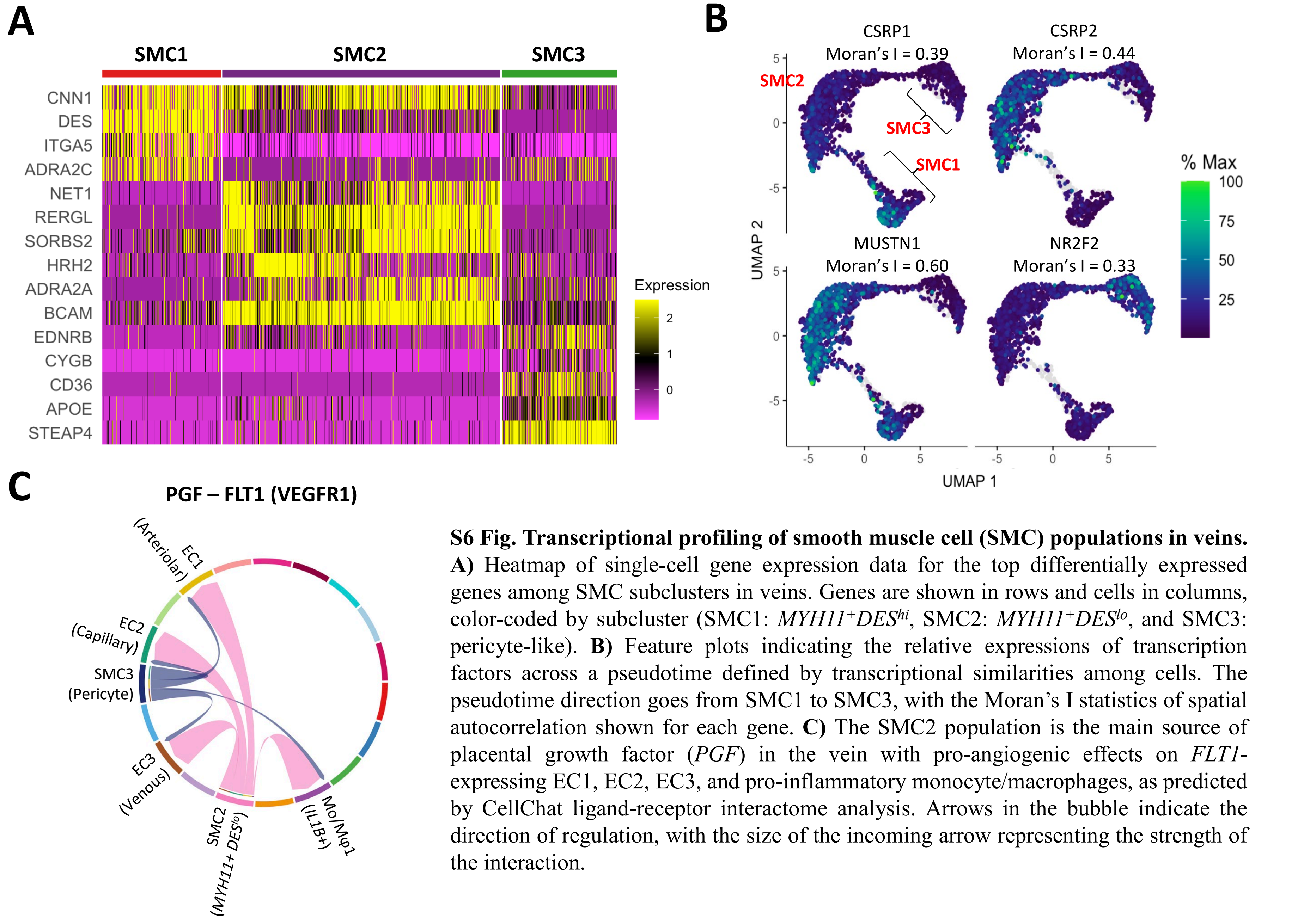

S6 Fig. Transcriptional profiling of smooth muscle cell (SMC) populations in veins. **A)** Heatmap of single-cell gene expression data for the top differentially expressed genes among SMC subclusters in veins. Genes are shown in rows and cells in columns, color-coded by subcluster (SMC1: *MYH11*$^{+}$*DES*^{hi}, SMC2: *MYH11*$^{+}$*DES*^{lo}, and SMC3: pericyte-like). **B)** Feature plots indicating the relative expressions of transcription factors across a pseudotime defined by transcriptional similarities among cells. The pseudotime direction goes from SMC1 to SMC3, with the Moran's I statistics of spatial autocorrelation shown for each gene. **C)** The SMC2 population is the main source of placental growth factor (*PGF*) in the vein with pro-angiogenic effects on *FLT1*-expressing EC1, EC2, EC3, and pro-inflammatory monocyte/macrophages, as predicted by CellChat ligand-receptor interactome analysis. Arrows in the bubble indicate the direction of regulation, with the size of the incoming arrow representing the strength of the interaction.
