# Supplementary material for: The intricate cellular ecosystem of human peripheral veins as revealed by single-cell transcriptomic analysis
Source: PLoS One. 2024 Jan 11;19(1):e0296264. doi: 10.1371/journal.pone.0296264 (PMC10783777; doi:10.1371/journal.pone.0296264)
Supplement: S7 Fig — A) Representative co-immunofluorescence of MYH11 and desmin in medial (left) and intimal SMCs (right). Cells with high (yellow-orange) and low desmin expression (green) coexist in both layers. B) Co-immunofluorescence of MYH11 and ITGA5 in medial (left) and intimal SMCs (right) shows similar distribution of ITGA5 high and low cells as in A. C) STEAP4+ pericytes surrounding microvessels. Scale bar = 20 μm in all panels. (PDF) [file pone.0296264.s008.pdf]

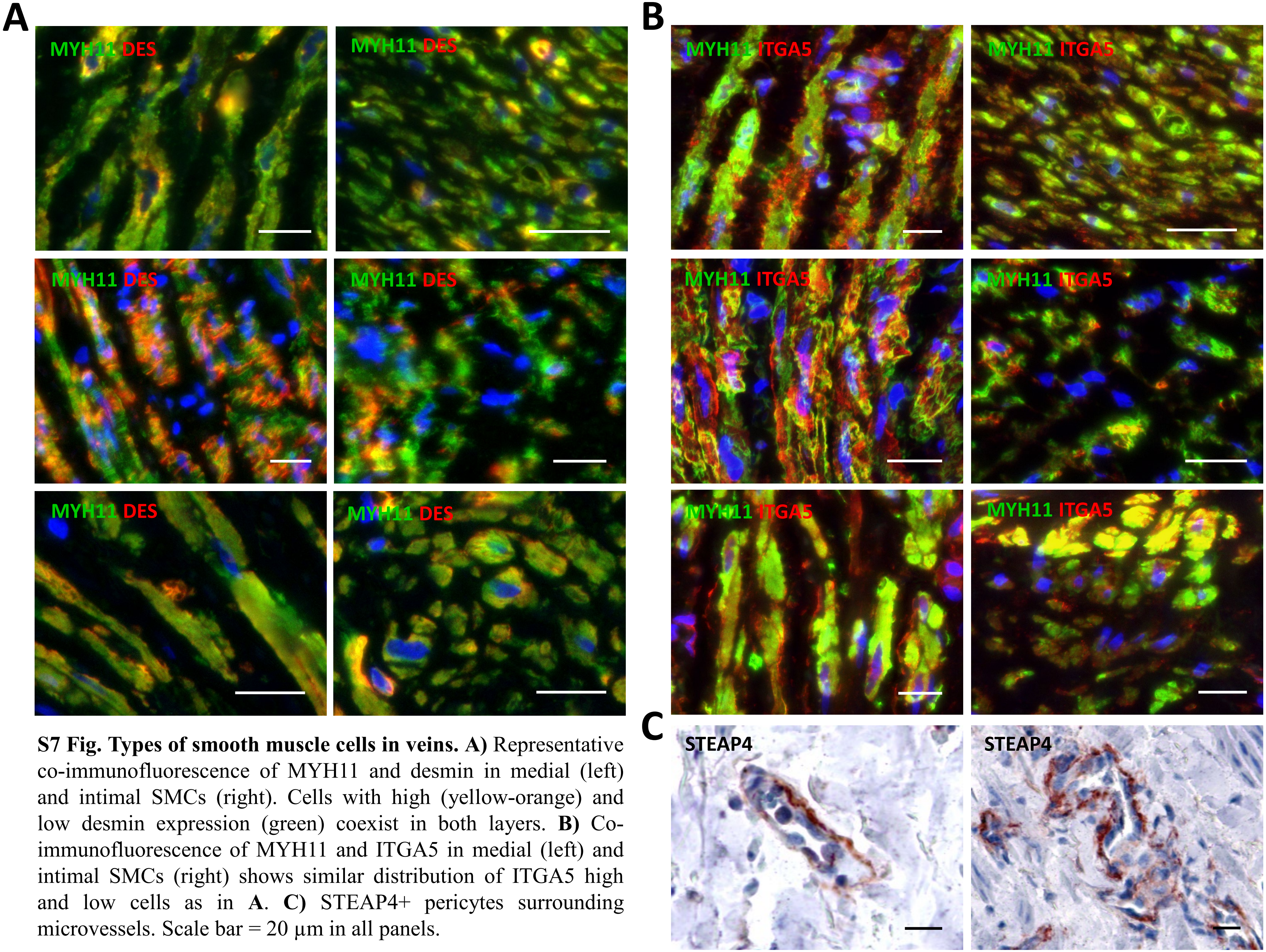

S7 Fig. Types of smooth muscle cells in veins. A) Representative co-immunofluorescence of MYH11 and desmin in medial (left) and intimal SMCs (right). Cells with high (yellow-orange) and low desmin expression (green) coexist in both layers. **B)** Co-immunofluorescence of MYH11 and ITGA5 in medial (left) and intimal SMCs (right) shows similar distribution of ITGA5 high and low cells as in **A**. **C)** STEAP4+ pericytes surrounding microvessels. Scale bar = 20 µm in all panels.
