# Supplementary material for: The intricate cellular ecosystem of human peripheral veins as revealed by single-cell transcriptomic analysis
Source: PLoS One. 2024 Jan 11;19(1):e0296264. doi: 10.1371/journal.pone.0296264 (PMC10783777; doi:10.1371/journal.pone.0296264)
Supplement: S8 Fig — A) Representative IHC of PDGFRA+ fibroblasts in veins demonstrates widespread distribution in the wall. Scale bars = 50 μm. B) Immunofluorescence of APOD in the intima (I), media (M), and adventitia (A). APOD+ fibroblasts appear in pink while extracellular APOD is shown in red. Scale bars = 20 μm. C) The Fib1 cluster is most closely related to SMC1 cells as predicted by pseudotime trajectory analysis. D) IGFBP5 in SMCs can potentiate the effects of insulin-like growth factor 1 (IGF1) from fibroblasts to promote SMC proliferation. (PDF) [file pone.0296264.s009.pdf]

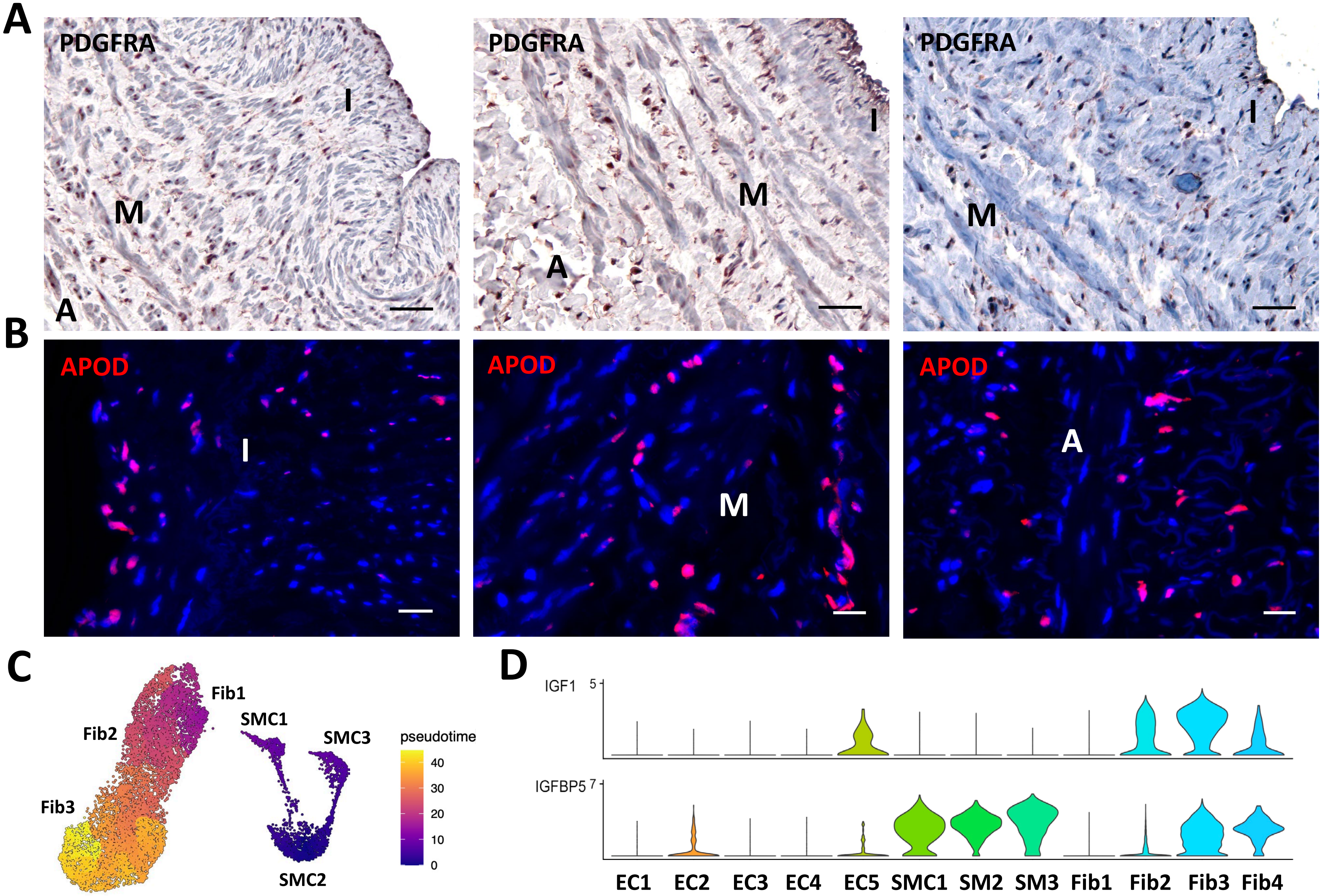

S8 Fig. Venous fibroblasts. A) Representative IHC of PDGFRA^{+} fibroblasts in veins demonstrates widespread distribution in the wall. Scale bars = 50 µm. **B)** Immunofluorescence of APOD in the intima (I), media (M), and adventitia (A). APOD^{+} fibroblasts appear in pink while extracellular APOD is shown in red. Scale bars = 20 µm. **C)** The Fib1 cluster is most closely related to SMC1 cells as predicted by pseudotime trajectory analysis. **D)** *IGFBP5* in SMCs can potentiate the effects of insulin-like growth factor 1 (*IGF1*) from fibroblasts to promote SMC proliferation.
