# Supplementary figures and images for: The intricate cellular ecosystem of human peripheral veins as revealed by single-cell transcriptomic analysis
Source: PLoS One. 2024 Jan 11;19(1):e0296264. doi: 10.1371/journal.pone.0296264 (PMC10783777; doi:10.1371/journal.pone.0296264)

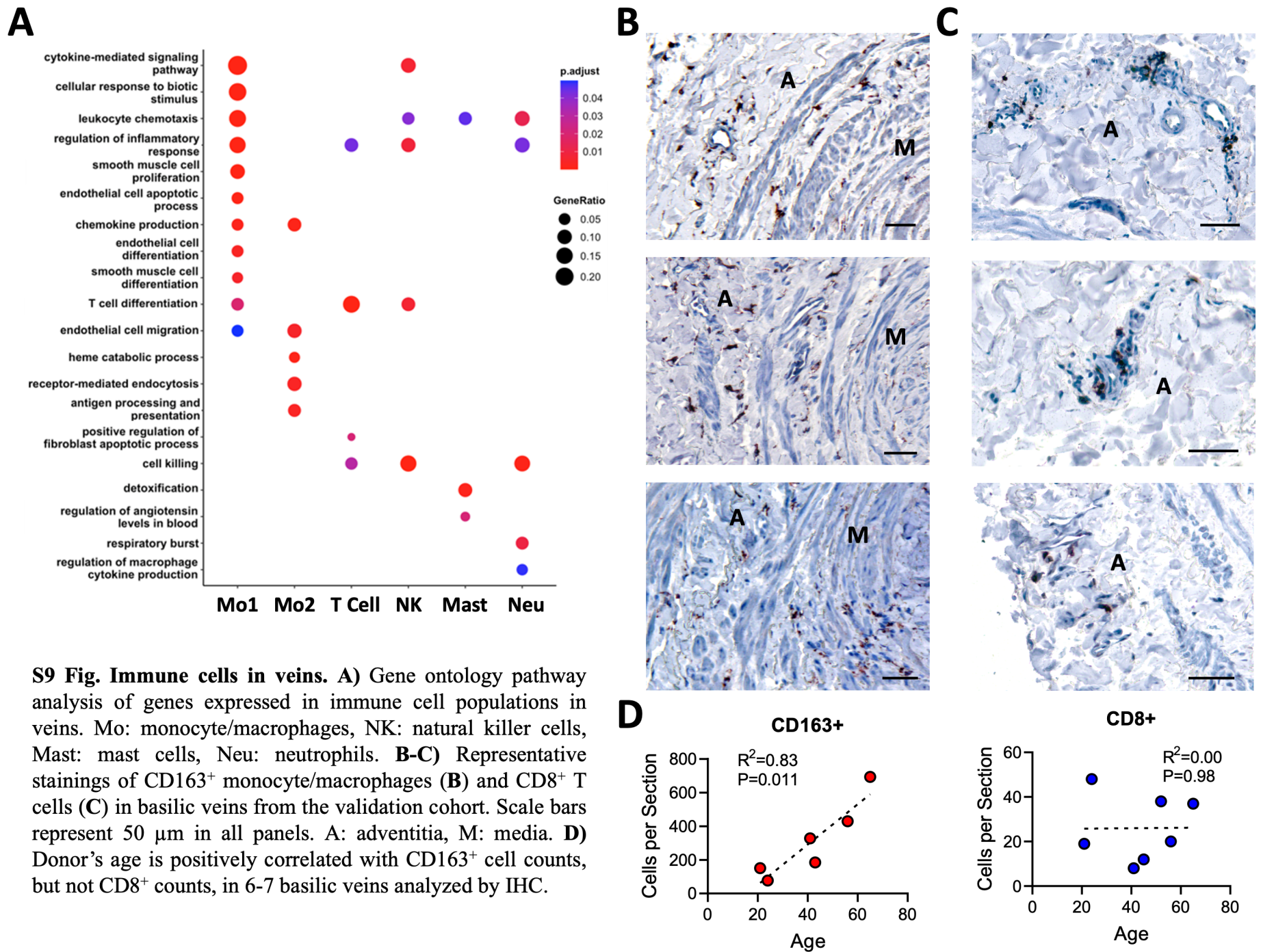

Supplement: S9 Fig — A) Gene ontology pathway analysis of genes expressed in immune cell populations in veins. Mo: monocyte/macrophages, NK: natural killer cells, Mast: mast cells, Neu: neutrophils. B-C) Representative stainings of CD163+ monocyte/macrophages (B) and CD8+ T cells (C) in basilic veins from the validation cohort. Scale bars represent 50 μm in all panels. A: adventitia, M: media. D) Donor’s age is positively correlated with CD163+ cell counts, but not CD8+ counts, in 6–7 basilic veins analyzed by IHC. (PDF) [file pone.0296264.s010.pdf]
