# Supplementary material for: The intricate cellular ecosystem of human peripheral veins as revealed by single-cell transcriptomic analysis
Source: PLoS One. 2024 Jan 11;19(1):e0296264. doi: 10.1371/journal.pone.0296264 (PMC10783777; doi:10.1371/journal.pone.0296264)
Supplement: S1 File — (PDF) [file pone.0296264.s011.pdf]

SUPPLEMENTARY METHODS

Study Subjects and Sample Collection

The study cohort included 16 organ donors whose tissues were donated for research through a collaboration with the Life Alliance Organ Recovery Agency (**S1 Table**). Cross-sectional samples of upper arm vessels (12 basilic veins, 3 cephalic vein, 1 brachial artery), approximately 2 cm in length, were obtained *post mortem* following organ procurement procedures. For single-cell RNA sequencing, 4 veins (3 basilic, 1 cephalic) were collected in cold DMEM/F12 (Gibco, Thermo Fisher Scientific, Waltham, MA) supplemented with 10% FBS (Sigma-Aldrich, St. Louis, MO), 1 mM sodium pyruvate, 100 U/mL penicillin, 100 ug/mL streptomycin, and 50 ug/mL gentamicin (all Gibco) for tissue dissociation. The remaining 12 samples were collected in RNA*later* (QIAGEN, Germantown, MD) and stored at -80°C. A 5 mm cross-section was fixed in 10% neutral formalin (Sigma-Aldrich, St. Louis, MO) for paraffin embedding and sectioning.

Tissue Processing

To generate single-cell suspensions for sequencing, veins were cut longitudinally with a sterile scalpel and then into 1-2 mm square sections. Samples were enzymatically digested for 90 minutes at 37°C with shaking using a combination of 3 mg/mL collagenase type II, 0.25 mg/mL soybean trypsin inhibitor, 0.2 mg/mL elastase, 1.4 U/mL Dispase, 60 U/mL DNAse I (all Worthington Biochemical, Lakewood, NJ), 0.15 mg/mL collagenase type XI, 0.25 mg/mL hyaluronidase type I, and 2.38 mg/mL HEPES (all Sigma-Aldrich) in HBSS with Ca^{2+} and Mg^{2+} (Thermo Fisher). After 90 minutes, an equal volume of Accumax (Sigma-Aldrich) was added to the dissociation mix and incubated for an additional 5 minutes. Single cells were filtered through

a 40 µM strainer, washed with HBSS twice, and incubated with red blood cell lysis buffer (BioLegend, San Diego, CA) for 5-10 min. Cells were washed once again with HBSS and resuspended in 0.1% BSA in PBS (Gibco) for counting and RNA sequencing.

Single-Cell RNA Sequencing and Alignment

Preparation of single-cell RNA libraries and sequencing were performed in the Center for Genome Technology at the University of Miami John P. Hussman Institute for Human Genomics. Single cell suspensions were counted using both the Cellometer K2 Fluorescent Viability Cell Counter (Nexcelom) and a hemocytometer. Samples with >80% viability were run using the Chromium Single Cell 3′ Library & Gel Bead Kit v3 (10X Genomics). The manufacturer's protocol was used with a target capture of 10,000 cells (5,000 for sample BV3). Each sample was processed on an independent Chromium Single Cell A Chip (10X Genomics), except sample BV2 which used two chips, and subsequently run on a thermocycler (Eppendorf). Sequencing libraries were evaluated for quality on the Agilent Tape Station (Agilent Technologies, Palo Alto, CA) and quantified using a Qubit 2.0 Fluorometer (Invitrogen, Carlsbad, CA) and qPCR before sequencing on the Illumina NovaSeq 6000. FASTQ files were generated with Cell Ranger's mkfastq pipeline (version 6.0.2). The Cell Ranger's count pipeline (version 6.0.2) was used to generate raw gene-barcode matrices from alignment to the 10X Genomics pre-built Cell Ranger human reference package (version 2020-A), from GRCh38 Ensembl build 98/GENCODE v32 gene annotations.

Bioinformatic Quality Control

Data from all 4 samples were processed in R (4.2.2) using Seurat v4 [89]. Briefly, cells with >15% mitochondrial content, >40% ribosomal content, <200 genes (nFeature_RNA), or predicted as doublets according to DoubletFinder [90] were filtered out from downstream analyses. We also adjusted for extracellular or ambient RNA using SoupX [91] as indicated in their vignette. Data were normalized using the "LogNormalize" method and using a scale factor of 10,000. Using Seurat's Scale.Data() function and "vars.to.regress" option, cell cycle, percent mitochondrial counts, and number of UMIs were used to regress out unwanted sources of variation. Unless noted otherwise, all bioinformatic packages were used as detailed in their respective vignettes with no major modifications to the R/Python Code.

Cell Clustering

On the merged Seurat object including all 4 veins, we applied the functions *NormalizeData*, *FindVariableFeatures*, *ScaleData*, and *RunPCA* using Seurat's standard parameters. We then removed batch effects and generated an integrated map using the package Harmony [92]. Overall clustering was performed with *FindNeighboors* and the first 30 principal components, followed by *Findclusters* and *RunUMAP* at a 0.5 resolution. We manually annotated the main clusters according to canonical cell markers. Clusters defined by markers of cell division (*CENPF, MKI67, TOP2A*) or with less than 40 cells were not included in downstream analyses.

Bioinformatic Inference Analyses

Pseudotime trajectory analysis of ECs, SMCs, and fibroblasts was performed as described in the Monocle3 vignette [93]. Briefly, we calculated a common trajectory for the connected

subpopulations using *learnGraph* and found the main transcripts that positively or negatively correlated with the trajectory using *principalGraphTest* to obtain the Moran's I coefficient of autocorrelation. Genes with padj <0.01 were considered as significantly correlated with the pseudotime cellular trajectory. Overrepresentation pathway analysis of differentially expressed genes (log_2FC >1 in Wilcoxon rank sum test and Min% expressed > 20%) was performed using the package clusterProfiler [94]. Biological Process (BP) pathways with padj < 0.05 using the function *compareCluster* were considered overrepresented in each subcluster. Ligand-receptor interactomes were analyzed using CellChat [95]. We imported the clustering metadata and generated a CellChat object with all subclusters following the package vignette. Overrepresented interactions were calculated using *identifyOverExpressedInteractions* and only those present in at least 20 cells per cluster were retained for downstream analysis.

Histology and Immunostaining

Tissue sections were stained with Masson's trichrome (#25088-1, Polysciences, Warrington, PA) and Movat's pentachrome (#ab245884, Abcam, Waltham, MA) for gross histomorphometric analysis. For immunohistochemistry, paraffin sections were rehydrated by serially immersing them in xylene, alcohol, and water, and antigens retrieved by boiling slides in 10 mM citrate buffer, pH 6.0 or Tris-EDTA buffer, pH 9.0 for 30 minutes (see table below). Sections were incubated with DAKO Peroxidase Blocking Solution (#K4005, Agilent, Santa Clara, CA) for 10 min, followed by DAKO Protein Blocking Solution (#X0909) for 1 hour. Specific proteins were detected by incubating overnight at 4°C with primary antibodies and dilutions as indicated below. Bound antibodies were detected using the DAKO EnVision System HRP labeled polymer anti-rabbit (#K4003) or anti-mouse (#K4001) secondary antibodies, or the ImmPRESS HRP

horse anti-goat IgG Polymer Detection Kit (#MP-7405, Vector Laboratories, Newark, CA). Color was developed with the DAKO AEC+ Substrate Chromogen System (#K4005). Nuclei were counterstained with Meyer's hematoxylin and mounted in VectaMount AQ Mounting Medium (#H-5501, Vector Laboratories). Images were acquired using a VisionTek DM01 digital microscope (Sakura Finetek, Torrance, CA).

For immunofluorescence, after antigen retrieval as above, sections were treated with 3% hydrogen peroxide and DAKO Protein Blocking Solution (#X0909), followed by primary antibodies diluted in DAKO Antibody Diluent Solution (#S3022) overnight at 4ºC. Bound antibodies were detected with Alexa Fluor 546 goat anti-rabbit antibody (1:1000, #A11081; all secondary antibodies from Thermo Fisher Scientific), Alexa Fluor 633 goat anti-mouse antibody (1:1000, #A21052), Alexa Fluor 546 donkey anti-mouse antibody (1:1000, #A10036), or Alexa Fluor 633 goat anti-rabbit antibody (1:1000, #A21071) for 45 minutes. Sections were counter-stained with 300 nM DAPI solution (#D1306, Thermo Fisher Scientific) in PBS for 5 minutes, and mounted in DABCO antifading polyvinyl alcohol mounting medium (#10981, Sigma-Aldrich). For signal amplification, after overnight incubation with primary antibodies, slides were incubated with biotinylated swine anti-rabbit polyclonal antibodies (1:1000, #E0353, DAKO) or biotinylated goat anti-mouse (1:1000, #OS02B, Oncogene Research) for 1 hour, then streptavidin HRP (1:1000, #P0397, DAKO) for 30 minutes, and finally amplified with a Tyramide Signal Amplification kit (1:50, #NEL700A001KT, Perkin Elmer, Waltham, MA). Amplification of the biotinylated antibody was detected through incubation with a streptavidin conjugated Alexa 546 secondary (1:1000, #S11029, Thermo Fisher Scientific) for 1 hour. Sections were examined in a Keyence All-in-One Fluorescence Microscope BZ-X800L and photographed using the Keyence BZ-X800 Viewer software.

TARGET	ANTIBODY	DILUTION	ANTIGEN RETRIEVAL
ACKR1	Abcam #ab137044,	1:50, IF (amplified)	pH 9
APOD	Invitrogen #PA5-27388	1:250, IHC or 1:100, IF	pH 9
CD8	R&D Systems #MAB3801	1:100, IHC	pH 9
CD163	Novus Biologicals #NBP2-48846	1:1000, IHC	pH 6
DES	DAKO #M0760	1:50, IF (amplified)	pH 9
GJA5	Invitrogen #37-8900	1:100, IF	pH 9
ITGA5	Sigma-Aldrich #HPA002642	1:100, IF	pH 9
MYH11	Novus Biologicals #NBP2-66967	1:50, IF	pH 9
PDGFRA	R&D Systems #AF-307-NA	1:200, IHC	pH 9
PLVAP	Invitrogen #PA5-51698	1:100, IF	pH 9
STEAP4	Proteintech #11944-1-AP	1:250, IHC	pH 9
